# Cancer Drug Response Profile scan (CDRscan): A Deep Learning Model That Predicts Drug Effectiveness from Cancer Genomic Signature

**DOI:** 10.1038/s41598-018-27214-6

**Published:** 2018-06-11

**Authors:** Yoosup Chang, Hyejin Park, Hyun-Jin Yang, Seungju Lee, Kwee-Yum Lee, Tae Soon Kim, Jongsun Jung, Jae-Min Shin

**Affiliations:** 1 Yongin in silico Medical Research Centre, Syntekabio Inc., 283 Dongbaekjungang-ro, C508, Giheung-gu, Yongin, Gyeonggi-do 17006 South Korea; 2 Gwanghwamun Medical Study Centre, Syntekabio Inc., 92 Saemunan-ro, #1708, Jongno-gu, Seoul, 03186 South Korea; 3 Faculty of Medicine, University of Queensland, Brisbane, QLD 4072 Australia; 4 Department of Clinical Medical Sciences, Seoul National University College of Medicine, 71 Ihwajang-gil, Jongno-gu 03087 Seoul, South Korea; 5 Genome Data Integration Centre, Syntekabio Inc., 187 Techno 2-ro, B512, Yuseong-gu, Daejeon, 34025 South Korea

## Abstract

In the era of precision medicine, cancer therapy can be tailored to an individual patient based on the genomic profile of a tumour. Despite the ever-increasing abundance of cancer genomic data, linking mutation profiles to drug efficacy remains a challenge. Herein, we report Cancer Drug Response profile scan (CDRscan) a novel deep learning model that predicts anticancer drug responsiveness based on a large-scale drug screening assay data encompassing genomic profiles of 787 human cancer cell lines and structural profiles of 244 drugs. CDRscan employs a two-step convolution architecture, where the genomic mutational fingerprints of cell lines and the molecular fingerprints of drugs are processed individually, then merged by ‘virtual docking’, an *in silico* modelling of drug treatment. Analysis of the goodness-of-fit between observed and predicted drug response revealed a high prediction accuracy of CDRscan (R^2^ > 0.84; AUROC > 0.98). We applied CDRscan to 1,487 approved drugs and identified 14 oncology and 23 non-oncology drugs having new potential cancer indications. This, to our knowledge, is the first-time application of a deep learning model in predicting the feasibility of drug repurposing. By further clinical validation, CDRscan is expected to allow selection of the most effective anticancer drugs for the genomic profile of the individual patient.

## Introduction

Over the past two decades, we have witnessed remarkable progress in understanding the complexity of genomic landscape of cancer. Exhaustive catalogues of somatic mutations of various cancer types have been created^1,2^, and major cancer-causing mutations have been identified^3^. It is not surprising that the expectation towards tailoring cancer treatments to a particular genomic signature of individual tumours is growing rapidly; yet, the current rate of new cancer agents to be approved and used clinically is deemed unsustainable for all stakeholders of healthcare, including cancer patients and pharmaceutical industry^4^. An efficient and systematic approach to evaluate the link between genomic information and the response of anticancer agents is very much needed.

Several collaborative efforts have been made to catalogue molecular profiling data of cancer cell lines and drug sensitivity data^5–7^ aimed at identifying genomic biomarkers predictive of anticancer drug response. Genomics in Drug Sensitivity in Cancer^6^ (GDSC, https://www.cancerrxgene.org) is an example of such publicly available databases providing experimentally measured drug sensitivities of 1,001 human cancer cells against 265 anticancer compounds^6^. Importantly, the molecular profiles of the entire cancer cell lines used in GDSC^6^ were extensively characterised as a part of COSMIC cell line project^1^ (CCLP, https://cancer.sanger.ac.uk/cell_lines), including profiling of somatic genomic alterations. These resources are expected to bring great benefit to the realisation of genomic-driven precision cancer medicine. Despite the potential value of such databases, the high-dimensionality and complexity of the datasets poses problems for integrative analysis. A number of approaches have been previously devised for the systematic identification of molecular markers in anticancer drug sensitivity using various machine learning methods. Examples of the methods include regularised regression, elastic net, random forests, kernel based methods, and/or shallow neural network^5,8–16^. However, these methods have their prediction accuracy at a moderate level at best and are often challenged by large-scale and highly intricate genomic data.

An increasingly applied method to enhance the prediction accuracy and to address the technical challenges is a deep learning model, which has received a great deal of attention with recent advances in information technology^17^. The deep learning method is a branch of machine learning techniques similar to shallow neural network but with multiple hidden layers and more complex parameters used in training. The method enables high level abstraction from a large volume of heterogeneous and high-dimensional raw datasets^18^. Until recently, the efficacy of learning was directly limited to the availability of relevant data^19^. Nevertheless, with a methodological improvement and a powerful machine with parallel computing horsepower, a deep learning model can be trained with multiple hidden layers, containing thousands of hidden units^20,21^. Since it can operate several types of structural information, such as pharmacological, genomic, and transcriptomic data, it is suitable for predicting drug-target interaction with minimal guidance^17^.

The pharmaceutical industry has begun showing its vested interest in deep learning to exploit these types of data for drug discovery^22^. Recently, several promising results have been demonstrated using deep learning in drug development^23–26^, drug-target profiling^27^ and drug repurposing/repositioning (i.e., identification of potential new purposes of approved or investigational drugs) with superior prediction accuracy compared to other conventional machine learning models^28,29^. Nevertheless, majority of the deep learning-based drug development focuses on the prediction of drug-target interaction, which is based on molecular structures, and few studies take genomics into consideration in developing their deep learning models^13^.

In this study, we have developed the Cancer Drug Response profile scan (CDRscan), a cancer genomic landscape-guided drug response prediction algorithm. By employing a novel dual convergence architecture deep learning model run on accelerated computing, and incorporating comprehensive drug response assay datasets obtained from CCLP^1^ and GDSC^6^ databases, the prediction accuracy of CDRscan was further enhanced over that of the previous computational modelling approaches. This accurate and robust drug response prediction model represents an important milestone for the realisation of precision cancer medicine through its application in the drug development processes such as drug repurposing and screening small chemical libraries for new anticancer drug candidate. In the clinical settings, CDRscan is expected to streamline patient-tailored anticancer drug selection as a clinical decision support system with further clinical validation studies.

## Results

### Overview of CDRscan and structure of datasets

**Figure 1:**
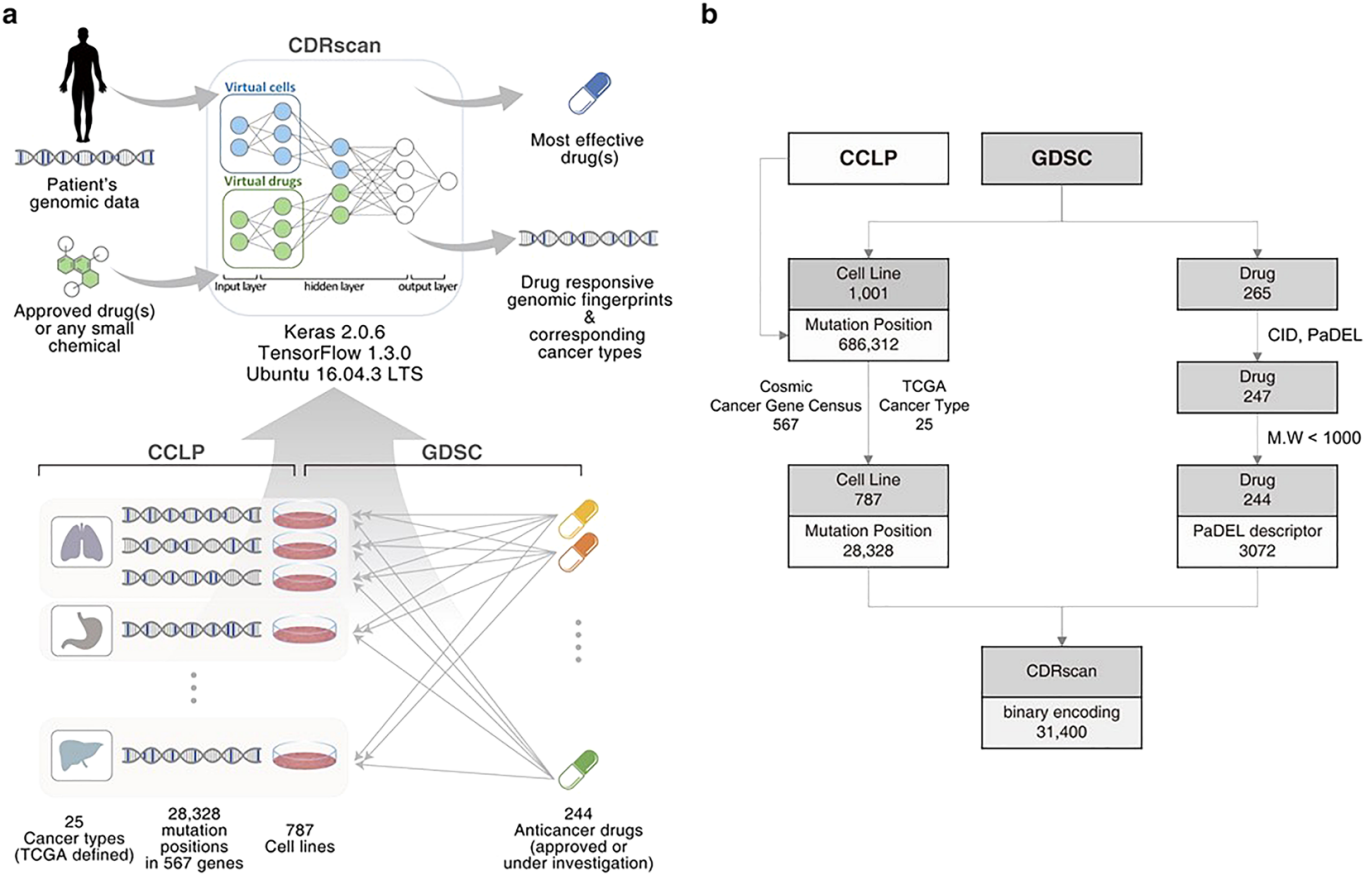
Overview of Cancer Drug Response profile scan (CDRscan). (**a**) Two main applications of CDRscan and dataset structure. For any given genomic fingerprint (i.e., a list of somatic mutations) of a tumour, CDRscan predicts which of 244 Genomics in Drug Sensitivity in Cancer (GDSC) anticancer drugs would be effective. The input of CDRscan can be molecular information of a particular small molecule for which CDRscan reports the predicted sensitivity of 787 cancer cell lines. The datasets used to train CDRscan were extracted from COSMIC cell line project (CCLP) and GDSC databases which represent 787 cancer cell lines across 25 cancer types defined by TCGA, 28,328 mutation positions in 567 cancer associated genes, and assay results from treatment of 244 anticancer drugs. (**b**) Data filtering procedure and final datasets. CCLP and GDSC databases contain genomic characterisation of 1,001 cancer cell lines and IC_50_ values measured from treatment of 1,001 cell lines with 265 anticancer drugs. The datasets were refined to include only the 567 Cosmic Cancer Gene Census genes and the cancer types that have at least 10 cell lines. Drugs without PubChem Compound Identifier or having molecular weight greater than 1000 g/mol were excluded. Totals of 28,328 and 3,072 features were extracted from cell line genomic signatures and drugs, respectively, constituting binary encoding of 31,400 features in total. The graphical image used in Fig. 1a is an original creation by Ye-Bin Jung and is reprinted under a CC BY license with permission from Ye-Bin Jung. All rights reserved.

CDRscan is an ensemble of five convolutional neural network (CNN)-based models^30^ with varied architectures (Table 1, Supplementary Fig. S1, Supplementary Table S1). Each model predicts the half-maximal inhibitory concentration (IC_50_) values of anticancer compounds from the genomic signature of tumour samples, and the mean is reported as the final prediction value. Input datasets were obtained from two perhaps the most comprehensive public databases related to cancer: CCLP for genomic profiles of human cancer cell lines^1^; and GDSC for anticancer drug sensitivity assays^6^ (Fig. 1a). The entire datasets from the databases contained 686,312 mutation positions from 1,001 cell lines and 265 drugs (Fig. 1b), covering 30 cancer types as defined by The Cancer Genome Atlas (TCGA) studies (https://gdc.cancer.gov/resources-tcga-users/tcga-code-tables/tcga-study-abbreviations). We curated a subset of the data to include only gene mutations contained in Cancer Gene Census^1,31^ (CGC, https://cancer.sanger.ac.uk/census), which is a catalogue of 567 genes strongly associated with cancer pathology (Fig. 1b, Table 2). In addition, the datasets were further screened to exclude the followings: (1) cancer types that were represented by fewer than 10 different cell lines (Table 2, Supplementary Table S2); (2) drugs without PubChem Compound Identifier; (3) drugs with molecular weight greater than 1000 g/mol (Supplementary Table S3, see Methods for more details on exclusion criteria). The final datasets yielded a total of 152,594 instances which contained 787 cell lines across 25 TCGA cancer types, mutation information at 28,328 base positions in 567 genes, and IC_50_ measurements of cell line-drug treatment in 244 drugs (Table 2, Supplementary Tables S2, S3).

**Table 1:** Five models of Cancer Drug Response profile scan (CDRscan).

**Model**		Virtual docking	Layer	Dense Activation	Compile Optimizer	Parameter	Epoch
**No**.	Name						
1	master	Yes	28	linear	Adam	365,486	250
2	fully connected	Yes	31	linear	Adam	367,116	500
3	shallow	Yes	24	linear	Adam	8,427,949	250
4	tanh	Yes	31	tanh	Adam	367,124	250
5	unified	No	17	linear	Adam	262,306	250

**Table 2:** Overview of genomic signature datasets.

Cancer Type (TCGA-defined)			Cancer gene census		
Abbreviation		Description	Number of cell lines	Number of genes	Number of mutation positions
**Datasets included in the final model**					
1	ALL	Acute lymphoblastic leukemia	26	441	2,104
2	BLCA	Bladder Urothelial Carcinoma	19	240	484
3	BRCA	Breast invasive carcinoma	51	372	1,103
4	CESC	Cervical squamous cell carcinoma and endocervical adenocarcinoma	14	219	406
5	COAD_READ	Colon adenocarcinoma and Rectum adenocarcinoma	51	478	5,582
6	DLBC	Lymphoid Neoplasm Diffuse Large B-cell Lymphoma	35	325	933
7	ESCA	Esophageal carcinoma	35	334	880
8	GBM	Glioblastoma multiforme	36	247	533
9	HNSC	Head and Neck squamous cell carcinoma	42	299	729
10	KIRC	Kidney renal clear cell carcinoma	32	233	490
11	LAML	Acute Myeloid Leukemia	28	326	816
12	LCML	Chronic Myelogenous Leukemia	10	214	346
13	LGG	Brain Lower Grade Glioma	17	176	285
14	LIHC	Liver hepatocellular carcinoma	17	181	315
15	LUAD	Lung adenocarcinoma	63	389	1,689
16	LUSC	Lung squamous cell carcinoma	15	188	324
17	MESO	Mesothelioma	21	170	286
18	MM	Multiple Myeloma	18	205	351
19	NB	Neuroblastoma	32	267	512
20	OV	Ovarian serous cystadenocarcinoma	34	336	917
21	PAAD	Pancreatic adenocarcinoma	30	230	448
22	SCLC	Small Cell Lung Cancer	66	405	1,598
23	SKCM	Skin Cutaneous Melanoma	55	391	1,610
24	STAD	Stomach adenocarcinoma	24	336	910
25	THCA	Thyroid carcinoma	16	201	310
Subtotal			787	567	28,328
**Datasets excluded from the final model**					
1	ACC	Adrenocortical carcinoma	1	20	20
2	CLL	Chronic Lymphocytic Leukemia	3	29	33
3	MB	Medulloblastoma	4	47	52
4	PRAD	Prostate adenocarcinoma	6	259	454
5	UCEC	Uterine Corpus Endometrial Carcinoma	9	377	1,168

The input features of the entire instance were represented by 31,400 binary digits. Of these, 28,328 bits represented mutational status of 28,328 genomic positions in each of the 787 cell lines, while 3,072 bits encoded molecular profiles of the individual drugs generated by PaDEL-descriptors^32^ (Fig. 1b). To effectively process the two distinctive types of inputs, namely the genomic fingerprints of cancer cell lines and the molecular fingerprints of the drugs, a dual convergence architecture was employed in four models (‘master’, ‘fully connected’, ‘shallow’, and ‘tanh’). A series of convolutions was performed independently for each set, thereby generating ‘virtual tumour cells’ and ‘virtual drugs’, respectively. Subsequently, ‘virtual docking’ (i.e., *in silico* simulation of drug treatment to cells) took place to merge the two separate convoluted features, followed by additional rounds of convolution. In one of the five CDRscan models (‘unified’), a conventional approach was employed, where all 31,400 descriptors in the input layer were convoluted together as one entity. All five models generated predicted IC_50_ values across the 244 anticancer drugs for each virtual cell line as a final output layer of the models. The average of the five values predicted by each model was then reported as the final outcome of CDRscan.

### Training of CDRscan and assessment of prediction accuracy

**Figure 2:**
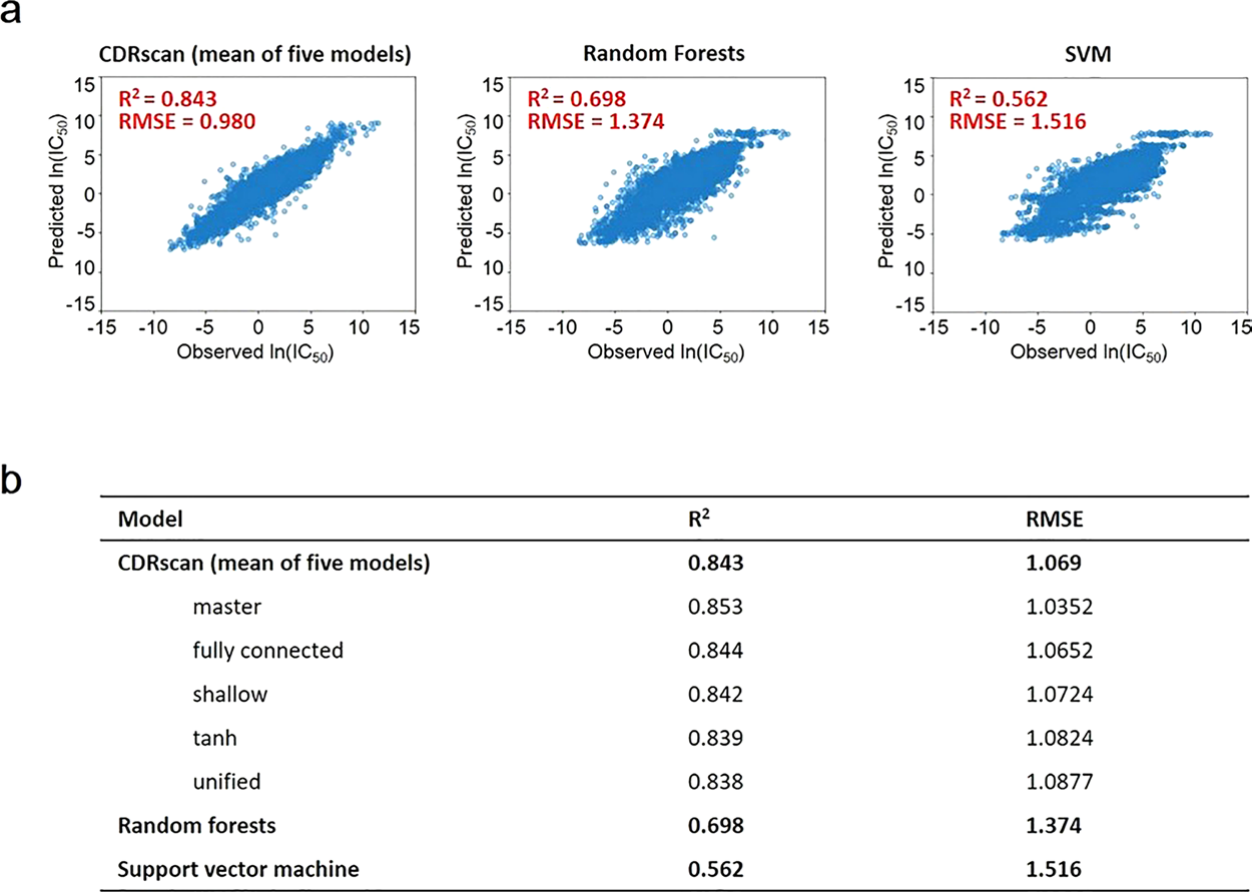
Assessment of prediction accuracy of CDRscan. (**a**) Scatter plots showing correlation between the observed and predicted IC_50_ values for CDRscan and two other machine learning models to benchmark the prediction accuracy. The test datasets, which correspond to 5% of the total cell line-drug pairs, were used to assess the coefficient of determination (R^2^). (**b**) Table summarizing the R^2^ values and root mean squared errors (RMSE) of CDRscan (mean value of the five models and values for individual models), random forest, and support vector machine.

Of the total of 152,594 instances spanning 25 cancer types, 144,953 instances (i.e., compilation of randomly selected 95% of instances for each cancer type) were selected to train all five models of CDRscan. The remaining 7,641 instances, corresponding to 5% of the total instances, were set aside for evaluation of performance of the models (test set). We then examined the correlation between the observed IC_50_ values from GDSC^6^ and the values predicted by CDRscan using the test set. The observed and the predicted IC_50_ values showed a strong agreement with the mean coefficient of determination (R^2^) value of 0.843, ranging from 0.838 to 0.853 across five models (Fig. 2a,b, Supplementary Fig. S2). Among the five models, the ‘master’ model exhibited the highest R^2^ (0.853). The four models that employed the dual convergence architecture had higher R^2^ values than the ‘unified’ model (Fig. 2b, Supplementary Fig. S2). The averaged root mean squared error (RMSE) value of the five models was 1.069 (s.d. = 0.018, n = 5), confirming that the prediction was accurate in most instances (Fig. 2b, Supplementary Fig. S2). To further confirm the prediction accuracy of CDRscan, we assessed the area under the receiver operating characteristic curve (AUROC) in the test set (n = 7,641). The cell lines were classified as being sensitive against a drug when ln(IC_50_) <−2 (−2 corresponds to IC_50_ of approximately 0.135 µM), which was set as a cut-off value for AUROC. The AUROC score of 0.98 was obtained as a result (Supplementary Fig. S3).

Across cancer types, a wide variation was demonstrated in the degree of mutation burden (Supplementary Fig. S4), in the total number of associated cell lines of the final datasets, and in the total number of mutation positions (Table 2). Nevertheless, when the performance of CDRscan was assessed against each cancer type, the predicted IC_50_ values for each of the 25 cancer types strongly agreed with the observed IC_50_ values across all models (Supplementary Fig. S5), wherein values of R^2^ ranged from 0.77 (chronic myelogenous leukaemia, LCML, n = 112) to 0.89 (lung squamous cell carcinoma, LUSC, n = 144) (Supplementary Fig. S5). Interestingly, ranks of 25 cancer types based on R^2^ values were identical across the five models (Supplementary Fig. S5). According to Pearson correlation analysis, we did not find any significant correlation between the prediction accuracy (i.e., R^2^ values) of individual cancer types and the average mutation burdens or the number of cell lines per cancer type (Supplementary Fig. S4).

**Figure 3:**
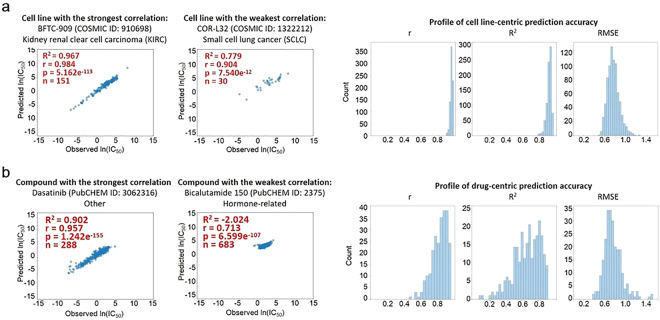
Cell line- and drug-centric correlation analyses. (**a**) Prediction accuracy assessment for each cell line. Scatter plots show the correlation between observed and CDRscan-predicted ln(IC_50_) values for the cell lines that showed the strongest (BFTC-909, left) and the lowest agreement (COR-L32, right). The COSMIC IDs of the two cell lines and the corresponding cancer types are indicated above the scatter plots, and the R^2^ values, Pearson correlation coefficient (r), p values, and the number of instances (n) are shown in the upper left corner of each plot. Histograms on the right show the overall distribution of prediction accuracy assessed for individual cell lines using indicated metrics. (**b**) Scatter plots showing the strongest and weakest agreement between observed and CDRscan-predicted ln(IC_50_) in drug-centric correlation analysis. The drug name and its PubCHEM ID are indicated in each plot. The R^2^ values, Pearson correlation coefficient (r), p values, and the instance counts (n) are also indicated. Histograms on the right show the overall distribution of prediction accuracy (R^2^) assessed for individual drugs using indicated metrics.

Lastly, the R^2^ values of individual cell lines (785 in total) and drugs (244 in total) were assessed. Two cell lines were excluded in the assessment since their drug treatment data was available for only one or two compounds, the number of which was too small to derive reliable R^2^ values. Cell line-centric analysis revealed a high mean R^2^ of five models between the observed and the predicted IC_50_ values for most cell lines. Amongst all cell lines, BFTC-909 (kidney renal clear cell carcinoma, KIRC) showed the highest R^2^ value of 0.967 (n = 151), while COR-L32 (small cell lung cancer, SCLC) had the lowest at 0.779 (n = 30) (Fig. 3a, Supplementary Fig. S6). Consistent with the high R^2^ values, the predicted and observed IC_50_ values showed strong correlation across all cell lines with p values less than 2.86e^−11^ (Fig. 3a).

In the drug-centric correlation analysis, dasatinib (tyrosine kinase inhibitor) had the highest mean R^2^ of 0.902 (n = 288), and bicalutamide (androgen receptor inhibitor) had the lowest at −2.024 (n = 683) (Fig. 3b, Supplementary Fig. S7). Drugs that had negative mean R^2^ values were characterised by small degrees of variance in the drug activity and in most cases showed little activity [ln(IC_50_) >−2] across all assayed cell lines. For instance, the observed ln(IC_50_) values of the bicalutamide-treated cell lines, having the lowest R^2^, ranged from −0.293 to 4.661 (mean = 2.586, range = 4.954), while those of dasatinib, the drug that was ranked the highest based on R^2^, ranged from −6.875 to 5.052 (mean = 0.222, range = 11.927) (Fig. 3b, Supplementary Fig. S7). When the Pearson correlation coefficient (r) was computed for all the drugs, even the drugs with low R^2^ values showed relatively high r values with p values less than 3.86e^−37^ (Fig. 3b).

One of the previous drug response prediction models has used the datasets obtained from the same databases as in our study (CCLP^1^ and GDSC^6^), although there were differences in the total number of datasets and in how the genomic and drug features were expressed^13^. To address whether and to what degree the application of dual convergence architecture deep learning model can improve the prediction accuracy, we compared the performance of CDRscan and the previously developed prediction model^13^. For direct comparison, we re-evaluated the prediction accuracy of CDRscan using multi-fold cross validation (five-fold with each fold containing 29,001 instances). The resulting R^2^ values of the five independent models of CDRscan ranged from 0.843 to 0.852 (Supplementary Table S4, Supplementary Fig. S8), which were comparable to that from the validation using the 5% leave-out test set (R^2^ = 0.843, Fig. 2a,b, Supplementary Fig. S2). Using the same cross validation method, we confirmed that CDRscan exhibited the performance that is significantly higher than that of the previous model (R^2^ = 0.72)^13^. Since the advent of the previous CCLP/GDSC-based machine learning model^13^, there has been a substantial increase in the amount of GDSC data sets^6^, which may also have influence the performance of the prediction model in addition to the deep learning architecture. We thus trained two commonly used machine learning models, random forest (RF) and support vector machine (SVM), using the identical instances to train (n = 144,953) and evaluate (n = 7,641) CDRscan, and compared the performances across the three models. The R^2^ values of RF and SVM models were 0.698 and 0.562, respectively (Fig. 2a,b), showing that their prediction accuracy is significantly compromised compared to CDRscan.

### Feasibility of drug repurposing using CDRscan

**Figure 4:**
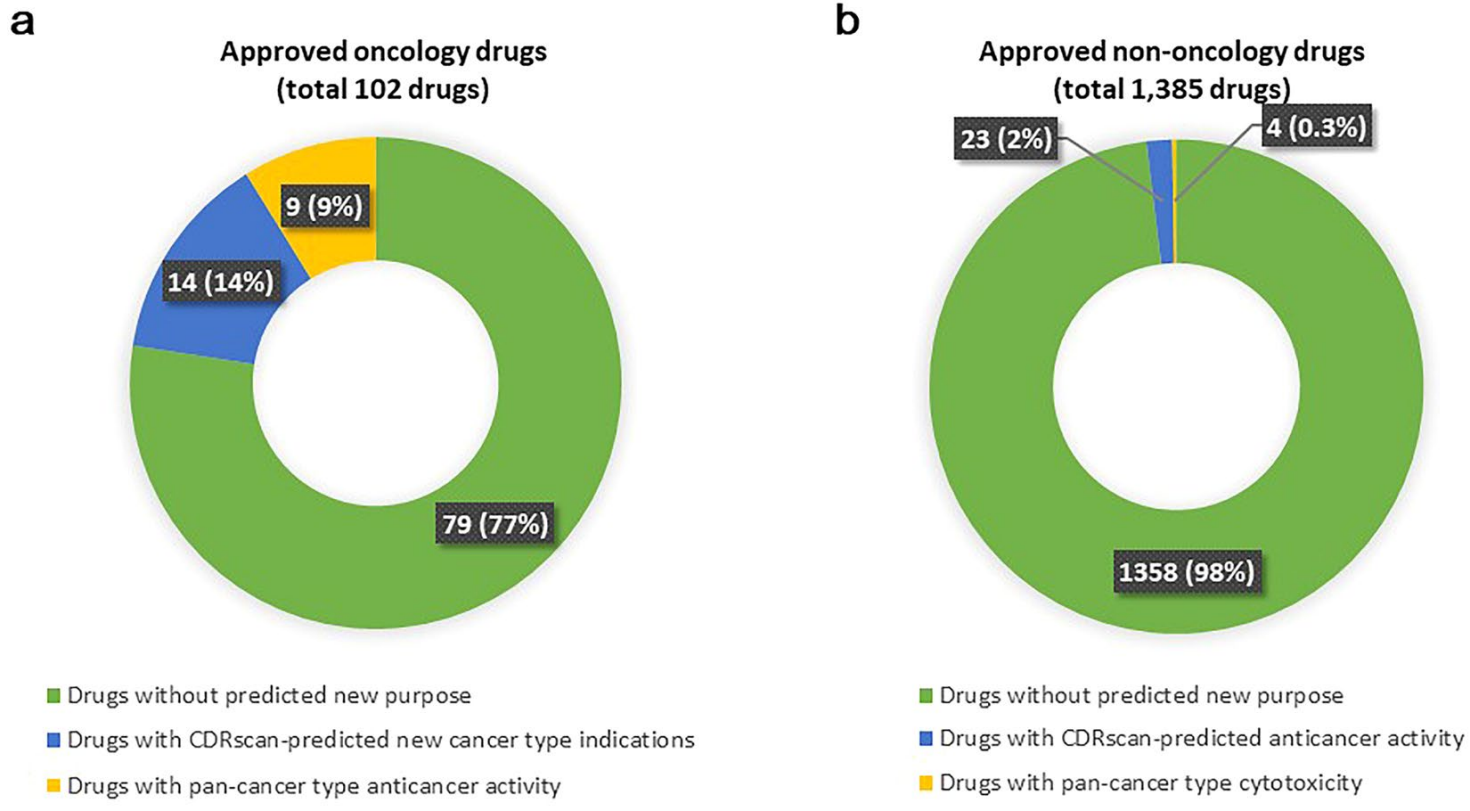
Feasibility of drug repurposing using CDRscan (**a**) Approved anticancer drugs with potential repurposing opportunity. CDRscan predicted that 23 out of 102 approved anticancer drugs have activity against at least one new cancer type in addition to the originally approved indications. Nine of these showed predictive sensitivity of more than 90% cancer types, indicating nonspecific antiproliferative/cytotoxicitc effects. (**b**) Approved non-oncology drugs with potential repurposing opportunity. Of the 1,385 non-oncology drugs, 27 showed potential anticancer activity. Four of these 27 drugs were predicted to have activity against over 90% of cancer types.

As a proof of concept in the drug repurposing potential of CDRscan, chemical descriptors of the approved drugs^33^ (see Methods for more details) were used to predict IC_50_ values of 787 cell lines. A total of 1,487 compounds were used in the analysis, and 102 of them were classified as oncology drugs according to National Cancer Institute^34^ (NCI, https://www.cancer.gov/about-cancer/treatment/drugs) (Fig. 4a). As in the AUROC analysis, ln(predicted IC_50_) < −2.0 was used to define positive anticancer drug response, which is a stringent criteria when compared to other similar studies^35^. Thirty seven of the 102 approved anticancer drugs had the potential for new caner type indications. In addition, 176 of 1,385 approved non-oncology drugs had the potential anticancer activities in addition to their original drug indications. However, some of the cancer types predicted as a new ‘purpose’ of the approved drugs contained only few drug responder cell lines. When those cancer types with only a few likely sensitive cell lines - less than 10% of the total cell lines showing predicted drug sensitivity - were excluded, the number of approved oncology drugs with repurposing potential was reduced from 37 to 23 (Fig. 4a). Nine of these 23 drugs had CDRscan-predicted anticancer activity against more than 90% (23/25) of the total types (Fig. 4a), suggesting a universal antiproliferative/cytotoxic activity of the compounds. Likewise, 27 of initially selected 176 non-oncology drugs (approximately 2% of 1,385 approved non-oncology drugs) showed strong predicted efficacy for at least one of the 25 cancer types, for which the predicted anticancer response was seen in at least 10% of the cell lines (Fig. 4b). Four of 27 drugs demonstrated strong predicted anticancer activity against more than 90% of all cancer types (Fig. 4b).

## Discussion

Here we describe CDRscan, a dual convergence architecture deep learning model, which predicts somatic mutation profile-based drug responsiveness by linking the tumour genomic fingerprint and its sensitivity to drugs. When the fingerprints of drugs or any small molecules are provided, CDRscan predicts the IC_50_ values of the CCLP panel of 787 human cancer cell lines (Fig. 1a). From these predictions, the new use of known drugs (i.e., drug repurposing) or novel anticancer agents can be inferred. Two major innovative methods were employed for the design of CDRscan, namely the dual convergence architecture for deep learning and the employment of five independent models. In the former, the cell line genomic fingerprint and the molecular fingerprint of drugs were modelled separately since they contained different types of information, and then the two were merged into one virtual docking model. With the latter, the robustness of the CDRscan was further enhanced by reporting the mean prediction values from multiple models. Of the five individual models of CDRscan, two models, ‘master’ and ‘fully connected’, performed better than the final ensemble model (i.e., mean prediction values). Nevertheless, by reporting the mean value from the ensemble of models, generalisability of CDRscan is ensured by minimizing the artefact that can be derived from a particular model architecture.

It has been shown that the best predictive outcome for estimating drug sensitivity can be obtained using gene expression data^36^, which has been taken into consideration for most of conventional computational modelling approaches^37^. However, genomic profile-based drug response prediction algorithms are in greater demand than of the gene expression-based algorithms, since genomic sequencing of a tumour sample is more commonly performed in clinics and for drug development. For instance, genetic testing for the detection of known biomarkers for targeted therapy is a routine clinical practice, and the majority of molecularly targeted cancer drugs are developed against cancer-specific mutant proteins^38^. The accurate prognosis and prediction of potential relapse by time series genomic analysis of cell free DNA may become a common practice in near future although it is currently costly and technically challenging^39,40^. We thus reasoned that development of a prediction model utilising genomic profile data including somatic mutations would have a wider array of practical application than a model using only gene expression profiles. Consistently high R^2^ values across all 25 cancer types in all five models strongly suggest that indeed the somatic mutation signatures have a significant influence on the responsiveness of anticancer drugs. Taken together, our model not only demonstrates high predictive accuracy and robustness, but also supports practicality for future applications.

Identification of new lead molecules for the treatment of diseases, including cancer therapies, is often the result of more than a decade of dedicated efforts from many research groups. Of these painstakingly selected drug candidates, only 5% finally make it to the market^41^. One solution to this issue is to use already approved drugs for new disease indications. By significantly reducing the time and cost associated with drug development^42^, drug repurposing benefits both patients and pharmaceutical industry. Despite the promise this method of drug discovery offers, currently there are limited success in repurposing drugs as anticancer agents mainly due to the lack of a systematic approach^43^. We propose that CDRscan can be an effective tool for this purpose by enabling high throughput *in silico* screening of drugs against their anticancer activity. When the chemical features of commercially available drugs are provided to CDRscan a subset of the 787 CCLP cell lines with potential sensitivity to the input drugs as well as the corresponding cancer types can be reported (Fig. 1a). The newly predicted cancer types represent a potential new indication for already approved drugs. Notably, CDRscan identified 37 approved drugs (2.5% of total 1,487 approved small molecule drugs in all therapeutic area) with potential new cancer indications. This number may be an underestimation since a stringent set of criteria were applied in our study to operationally define a drug with potential repurposing opportunity: (1) ln(IC_50_) <−2 for anticancer response; (2) 10% or more cell lines within the cancer type to have the predicted drug response; (3) exclusion of the drugs with pan-cancer type antiproliferative/cytotoxic effect. *In vitro* and/or clinical evidence supported a subset of the new, CDRscan-predicted indications of multiple oncology drugs (e.g., belinostat^44–47^, cabozantinib^48^, cobimetinib^49^, etc.) and non-oncology drugs (e.g., pravastatin^50–52^ and ouabain^53–55^). Notably, many of the potentially repurposable drugs with supporting evidence were not in GDSC (i.e., outside the CDRscan training set), strongly arguing for the prediction accuracy as well as the practicality of CDRscan as a systematic *in silico* screening method for drug repurposing. Although the predicted results require *in vitro* validation prior to subsequent clinical studies, our results offer a realistic opportunity in drug repurposing. It should also be emphasised that CDRscan can be utilised to screen for new candidates of lead molecules when the chemical features of small compounds from chemical libraries such as the ZINC^56,57^ are used as an input instead of limiting the input to approved drugs.

It is important to note that CDRscan predicts a drug response at the level of cancer cell lines, not of the cancer types. We considered the cancer types as new indications when they contained the susceptible cell lines with ln(predicted IC_50_) less than −2. This criteria was chosen for a practical reason since the current guideline for anticancer drug treatment is primarily based on the site of a tumour^22^. However, one should keep in mind that the genomic fingerprints of the predicted drug responder cell lines may not be truly representative of the cancer type that they belong to. It is important to evaluate how frequently drug response-predictive mutation patterns appear between the genomes of various cancer types. In a hypothetical scenario where a predictive mutational pattern is present in many cancer patients but independent of cancer types, new indication of the drug to be repurposed should be determined by genomic biomarkers, not by cancer types. Ideally, the identification of a minimal number of mutations that appear specifically in sensitive cell lines irrespective of cancer types would represent a good candidate of ‘genomic biomarker’ for indication. In line with this, for the first time, the U.S. Food and Drug Administration has recently approved Keytruda (pembrolizumab) for tumours with a certain genetic biomarker regardless of their origin^58^. The drug response-associated genomic markers have already been included in drug labels to serve as guidelines for drug prescription, and the genomic marker based approval of anticancer drug is expected to become a more common practice. The rise in the number of companion diagnostics for detecting specific genomic biomarkers is also expected to gain momentum.

Recent analyses have reported that only a limited number of cancer patients would benefit from genetic screening to select targeted drugs using known biomarkers, since those with positive biomarkers are rare^59,60^. Even among patients with a well-defined biomarker for targeted therapies, the expected clinical outcome is often lost due to prevalent crosstalk between genes. In the clinical settings, CDRscan can be applied to screen for the drugs with the likely effectiveness for individual tumours. As CDRscan predicts drug sensitivity from genomic fingerprints rather than individual mutation, it further broadens the use of genomic screening results in the identification of effective drugs. Eventually, CDRscan would facilitate individually tailored cancer treatment, guiding clinical decision in the selection of anticancer agent(s) that best suits the genomic signature of the individual patients.

Challenges still remain for routine application of CDRscan. Despite its strong predictive accuracy, the model was built on *in vitro* data. Cancer cell lines of CCLP^1^ and GDSC^6^ represent cancer models that are extensively used in the cancer research community^33,61–63^. While those databases archive a large collection of cancer cell lines across a broad spectrum of cancer types, it remains to be determined whether these cell lines truly represent tumours that are growing *in vivo*^41,64^. Cancer cell lines are derived from patient tumour samples, but they are homogenous, subject to additional mutational events, and not surrounded by microenvironment. Thus, further *in vivo* validation of the predictions made by CDRscan is a mandatory next step. Especially, given that CDRscan is developed using selected mutation positions significant for cancer (28,328 positions from 567 genes), mutations occurring outside this range is not used as inputs for genomic fingerprints. Expanding the input positions by coupling it with a solid analysis for clinical validation can be done in the future by using genomic sequence data of cancer patients receiving chemotherapy. In recent years, patient-derived organoids^65^ and xenograft mice^66^ have become widely accepted platforms for drug screening in cancer research. These new platforms open up additional avenues for validating the performance of CDRscan.

By incorporating additional data types into computational models, the predictive prowess can be further strengthened^15,33^. Among several types of molecular profile data, transcriptomes of a large cohort of cancer cell lines in particular have been extensively characterised by a few large-scale projects such as CCLP^1^ and Cancer Cell Line Encyclopedia^1,5^. In this regard, there is no doubt that the performance of CDRscan can be further improved by incorporating additional high quality ‘-omics’ data including transcriptome information. Despite the excellent overall performance of CDRscan, prediction accuracy of a few GDSC compounds was compromised with low R^2^ values due to limited variance of data where the observed IC_50_ values had a very narrow range (Fig. 3b, Supplementary Fig. S7). Although most of the drug-centric analysis of CDRscan showed strong prediction of trend/order with high r values, incorporating additional assay results whenever available would also enhance the accuracy of prediction.

In this study, we introduced CDRscan, a novel and robust ensemble of deep learning models to predict drug efficacy based on genomic signatures. A web service will soon be available to allow browsing of the datasets used in CDRscan, the predicted and observed IC_50_ values, and some of the interesting examples of newly predicted indications for approved drugs. With further improvement, we envision that CDRscan will contribute to the rapidly evolving field of oncology by promoting the use of genome data for precision cancer medicine.

## Methods

### Software and hardware

To design CDRscan, we have implemented CNN using TensorFlow 1.3.0, Keras 2.0.6., and Ubuntu 16.04.3 LTS. Model design, training, and validation were performed on a workstation equipped with NVidia GTX 1080Ti.

### Datasets

The genomic and drug sensitivity datasets of a wide array of cancer cell lines were obtained from CCLP^1^ (version 82, grch37) and GDSC^6^ (release 6.0). CCLP^1^ contains various types of molecular profile data, including whole exome sequencing data of 1,001 human cancer cell lines commonly used in cancer research. We selected sequence variation information at 28,328 positions from 567 genes in CGC^31^ (COSMIC v82, last obtained in Oct 2017). Five types of cancer (Table 2) were excluded since they had a limited number of available cell lines (<10). As a result, the final dataset contained 787 cell lines.

GDSC^6^ provides IC_50_ values from drug sensitivity assays for over 200,000 drug-cancer cell line pairs. In GDSC^6^, the identical set of 1,001 cell lines genomically characterised by CCLP^1^ was used, and 265 anticancer therapeutics from various sources, ranging from approved to those under investigation, were included in the assays. A line notation of simplified molecular-input line entry system (SMILES)^67^ was initially used to extract the structural and chemical features of each drug from PubChem^68^. However among 265 drugs, 18 drugs were not registered in SMILES, and three drugs had a molecular weight exceeding 1,000 g/mol. These 21 drugs were removed from the dataset. We also noticed that in GDSC, some identical chemicals were counted as two discrete entities^6^. There were 15 such pairs, but since the IC_50_ values were different across all pairs, we considered the 15 pairs as 30 distinctive drugs. The final dataset had 244 drugs representing 229 individual small chemicals (Fig. 1b, Supplementary Table S3). A total of 152,594 instances were in the final matrix of cell lines and drugs and employed to develop the deep learning models.

### Feature extraction

Two different sources of input were used in CDRscan, which were genomic sequence variations of individual cancer cell lines and chemical properties of the anticancer drugs. The genomic fingerprints of cancer cell lines were expressed as a string of 28,328 binary codes, each representing a somatic mutation status. We have used only the variants that passed a series of stringent quality control filters, which were finally accepted in the COSMIC database. The list of somatic mutations were filtered further to include only those in CGC^1^. The presence of a somatic mutation was encoded as 1 and absence as 0. In a given mutation position, the same position substituted with a different base was considered identical. Insertions and deletions were not distinguished from base substitutions.

For SMILES of each of the 244 GDSC drugs^6^, a PaDEL-descriptor (v2.2.1)^32^ was employed to extract descriptors of three classes of fingerprints: (1) fingerprinter, (2) extended fingerprinter, and (3) graph only fingerprinter^69–71^, totalling 3,072 binary descriptors per drug, termed ‘molecular fingerprints’.

### Model architecture

Two main principles were applied in designing CDRscan deep learning models. First, we employed novel CNN architectures, which we designated as a dual convergence deep learning architecture, to effectively process two very distinct types of input information. This architecture was applied to four of the CDRscan models. Second, we aimed to design a generalisable prediction model. To this end, we employed an ensemble of five independent models to minimise artifact derived from a particular model architecture. The architecture of each model was designed so that they were sufficiently distinct from one another. The major differences we introduced to the model architectures were as follows: (1) The number of convolution layers, (2) presence of absence of fully connected layer, (3) normalisation, and (4) inclusion of dual convergence architecture (Table 1, Supplementary Fig. S1, Supplementary Table S1).

One of the main hurdles for designing a robust deep learning model is overfitting. We thus employed the following techniques to prevent overfitting (Supplementary Fig. S1, Supplementary Table S1): (1) Three to four dropout layers were applied in all five models. In these layers, a subset of parameters (10–20% of the total parameters) were randomly selected and ignored during training, making it less likely to overfit the training data. (2) Maxpooling layers, which reduce dimensionality of input, were included in all models. When the mean square errors (MSE) of train and test set were plotted against the number of epochs, we did not observe an increase nor fluctuations of the test set error (Supplementary Fig. S2), which are considered as typical signs of overfitting. In addition, (3) the performance score of CDRscan measured by five-fold cross validation was consistent (described in the next section), indicating that the high performance score of CDRscan was not due to overfitting (Supplementary Table S4, Supplementary Fig. S8).

### Model training and performance evaluation

We randomly selected 144,953 instances to train the models (95% of the total 152,594 instances). To ensure that all 25 cancer types are represented equally in the training set, we randomly choose 95% of the instances from each cancer type. As a result, 25 subsets were created and subsequently compiled as a single training set. The remaining 5% of the instances of individual cancer types were set aside to be used as test sets (i.e., train-test split method), both as 25 separate lists and as one consolidated list. Next, the performance of CDRscan and its five individual models was evaluated using the test datasets. For the evaluation, the experimentally obtained (observed) IC_50_ values and their counterparts predicted by CDRscan were plotted on a natural log scale. A coefficient of determination (R^2^), a widely accepted measure of prediction accuracy in machine learning^72^, was then computed using the following formula:

$${{R}}^{2}=1-\frac{{\sum }_{{i}}^{{n}}{({{\rm{y}}}_{{i}}-{{f}}_{{i}})}^{2}}{{\sum }_{{i}}^{{n}}{({{\rm{y}}}_{{i}}-\bar{{y}})}^{2}}$$

where y_1_, y_2_, … y_n_ are observed IC_50_ values of each test set with n instances; f_1_, f_2_, …, f_n_ are the predicted IC_50_ values for the corresponding instances;$\,\bar{{y}}$ is the mean of the observed values.

Root mean squared error (RMSE) values were also calculated to evaluate the level of accuracy of prediction. Performance evaluation was also performed on the 25 separate test sets for individual cancer types, and for individual cell lines or for individual drugs. In the drug-centric evaluation of CDRscan performance, Pearson correlation coefficients (r) and p values were assessed for selected subsets of instances using SciPy (https://www.scipy.org; version 0.19.1). AUROC was also computed for the compiled training set. A classification criteria was applied to IC_50_ values using ln(IC_50_) of −2 as a cut-off (approximately 0.135 µM), where a drug is deemed active at ln(IC_50_) <−2 and inactive otherwise.

To benchmark the level of prediction accuracy, random forest and support vector machine models were trained with the identical test sets and parameters used in CDRscan. The prediction accuracy of two machine learning models were assessed using the same evaluation metrics.

In five-fold cross validation of CDRscan, the train set (144,953 instances) was partitioned into five equal-sized subsamples (28,991 instances in each subsample). One of the subsamples was held as a test set, and each model was trained with the remaining four subsamples. This process was repeated four more times, each time leaving a different subgroup as a test set.

### Demonstration of predicting potential new indication of already approved drugs using CDRscan

The list of drugs approved in U.S., Canada, or E.U. was obtained from DrugBank^33^ (https://www.drugbank.ca; last accessed on September 20^th^, 2017). This list contained small molecules as well as complex molecules, such as biologic drugs, which cannot be properly expressed by 3,072 PaDEL descriptors used in CDRscan. Thus, the drugs that are compatible for CDRscan were selected from the initial list of the approved drugs by excluding the compounds according to the following criteria: (1) having no SMILES and/or no molecular weight information; (2) having a molecular weight <200 g/mol or >650 g/mol; (3) containing two or more parts that are not bonded together (including ‘ . ’ in their SMILES); and (4) being inorganic compounds. As a result, the final list of 1,487 approved drugs was generated. The final list included both non-oncology and oncology drugs, in which the oncology drugs were from the list provided in National Cancer Institute (NCI, https://www.cancer.gov/about-cancer/treatment/drugs). The structure information of these drugs was converted into 3,072 descriptors using the aforementioned PaDEL-descriptor (v2.2.1), which were used as input for CDRscan. To define ‘drug-sensitive’ cell lines, ln(predicted IC_50_) of −2 was used again as a cut-off, and the cancer types were designated as being sensitive to a given drug when more than 10% of the corresponding cell lines had ln(predicted IC_50_) less than −2. When counting the number of the potentially novel anticancer drugs, all non-oncology drugs with at least one sensitive cancer type were counted. For oncology drugs, only those with CDRscan-predicted cancer type indications in addition to the originally approved ones were considered as the drugs with repurposing potentials.

## Electronic supplementary material

Supplementary Information
