# Supplementary material for: Cancer Drug Response Profile scan (CDRscan): A Deep Learning Model That Predicts Drug Effectiveness from Cancer Genomic Signature
Source: Sci Rep. 2018 Jun 11;8:8857. doi: 10.1038/s41598-018-27214-6 (PMC5996063; doi:10.1038/s41598-018-27214-6)
Supplement: Supplementary file 1 — Supplementary Information [file 41598_2018_27214_MOESM1_ESM.pdf]

Cancer Drug Response Profile scan (CDRscan): A Deep Learning Model That Predicts Drug Effectiveness from Cancer Genomic Signature

Yoosup Chang[1], Hyejin Park[1], Seungju Lee[1], Hyun-Jin Yang[2], Kwee-Yum Lee[2,3], Tae Soon Kim[2,4], Jongsun Jung[5]*, and Jae-Min Shin[1]*

[1]Yongin *in silico* Medical Research Center, Syntekabio Inc., 283 Dongbaekjungang-ro, C508, Giheung-gu, Yongin, Gyeonggi-do, 17006, South Korea, [2]Gwanghwamun Medical Study Center, Syntekabio Inc., 92 Saemunan-ro, #1708, Jongno-gu, Seoul, 03186, South Korea, [3]Faculty of Medicine, University of Queensland, Brisbane, QLD, 4072, Australia, [4]Department of Clinical Medical Sciences, Seoul National University College of Medicine, 71 Ihwajang-gil, Jongno-gu, 03087, Seoul, South Korea, [5]Genome Data Integration Center, Syntekabio Inc., 187 Techno 2-ro, B512, Yuseong-gu, Daejeon, 34025, South Korea

*Corresponding authors

Supplementary Figure S1. Architectures of individual models of CDRscan.

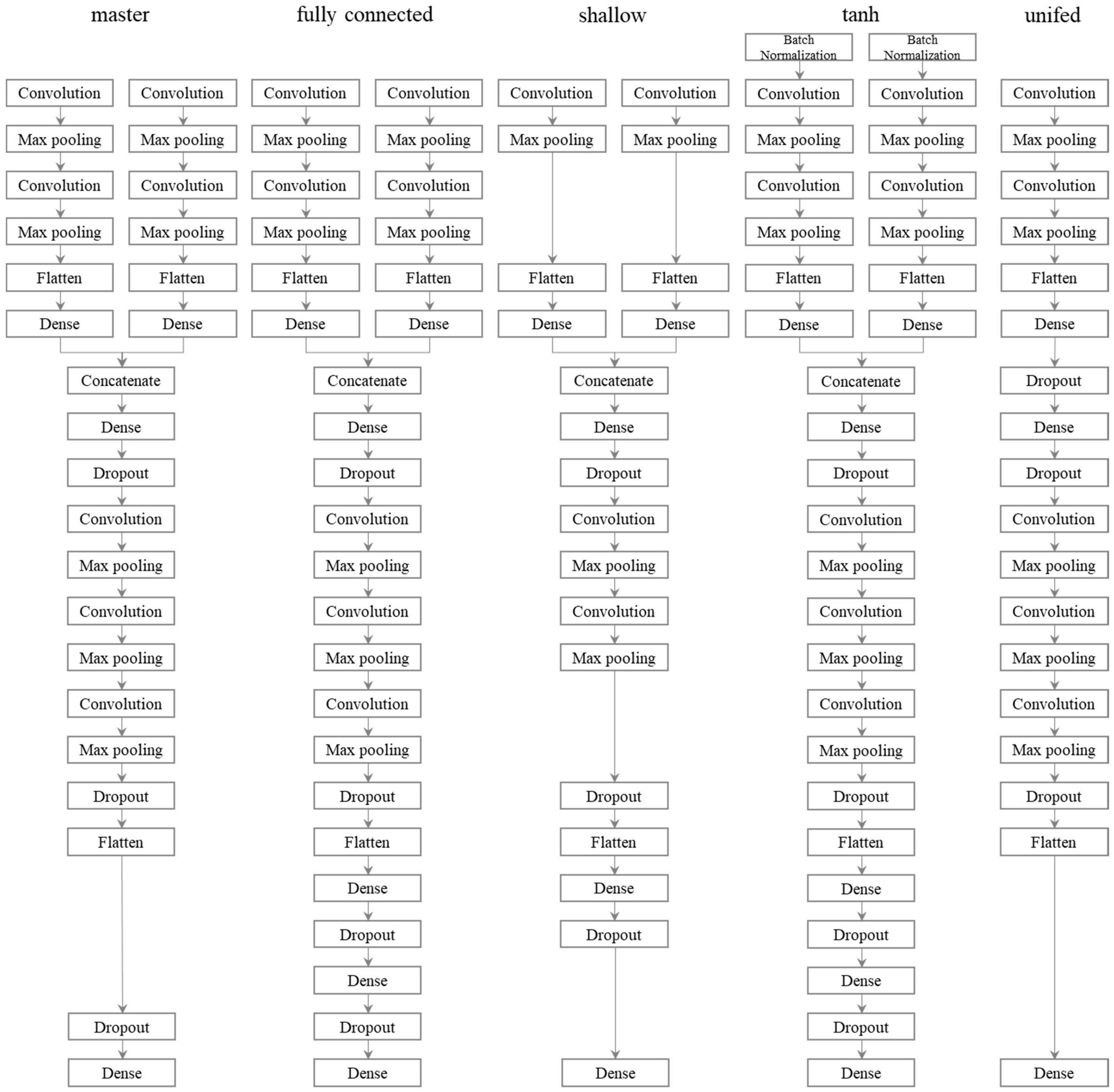

Architectures of individual models of CDRscan. To achieve generalizability of the prediction model and to avoid having model-specific artifact, we designed an ensemble model that is composed of five independent models. The major differences introduced to individual models include the number of the convolution layers, presence of absence of fully connected layer, normalisation, and inclusion of the novel dual convergence architecture.

Supplementary Figure S2. Assessment of prediction accuracy of individual CDRscan models.

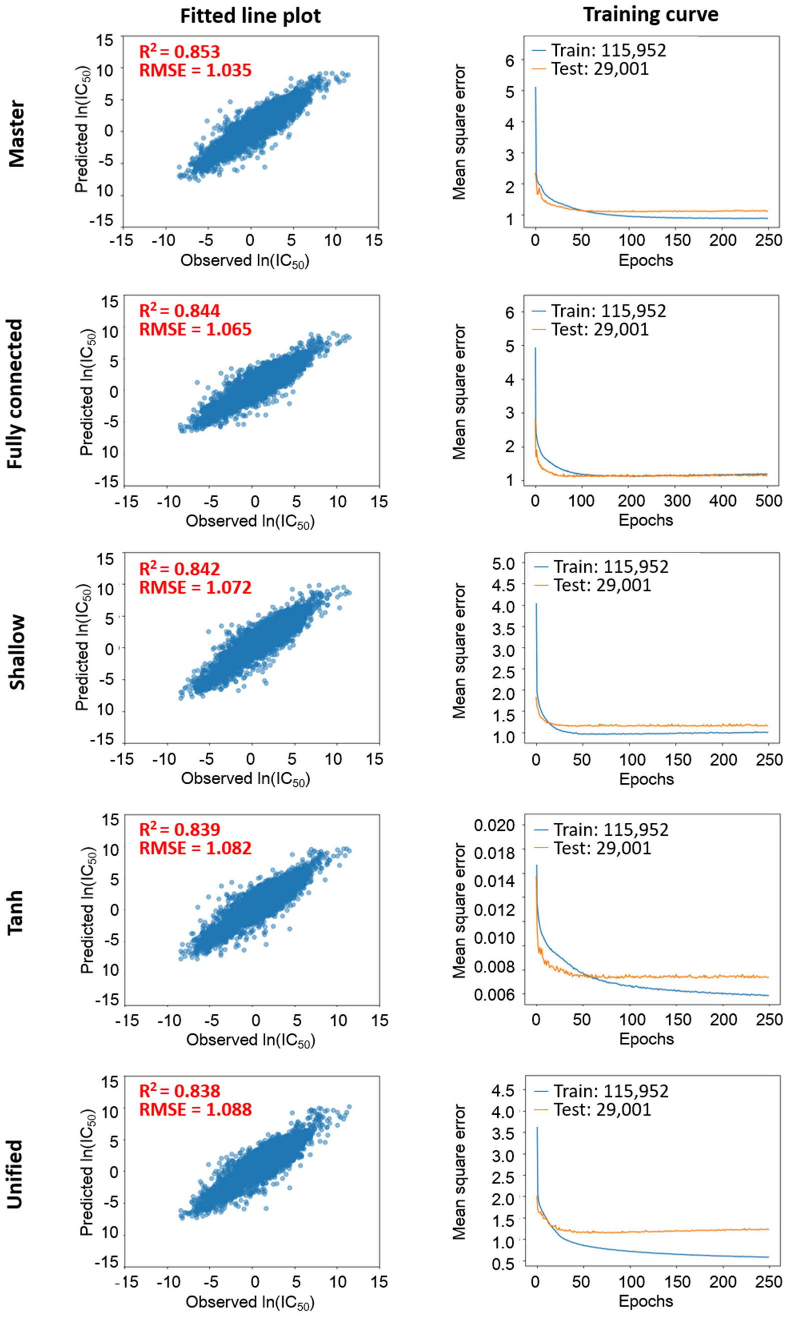

Performance of individual models of CDRscan was evaluated with 5% held out test set. Scatter plots show for the individual models the correlation between the observed and the predicted IC_{50} values (in a natural log scale). The R^2 values and root mean squared errors for each model were indicated. Training curves show mean squared errors of train (blue) or test (orange) set at each epoch. The number of data points used in the train and the test set was indicated.

Supplementary Figure S3. Assessment of prediction accuracy of CDRscan using AUROC.

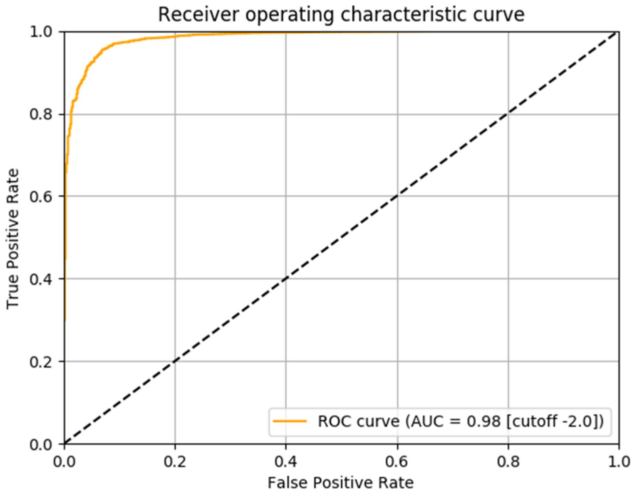

The area under the receiver operating characteristic curve (AUROC) was computed with a validation set (5% of all instances, which were not used for training, n = 7,641) using $lnIC_{50}$ of -2 as a cutoff. A drug was considered active for a given cell line when $lnIC_{50}$ was lower than -2 and considered inactive otherwise.

Supplementary Figure S4. Mutational status across all cell lines over 25 cancer types.

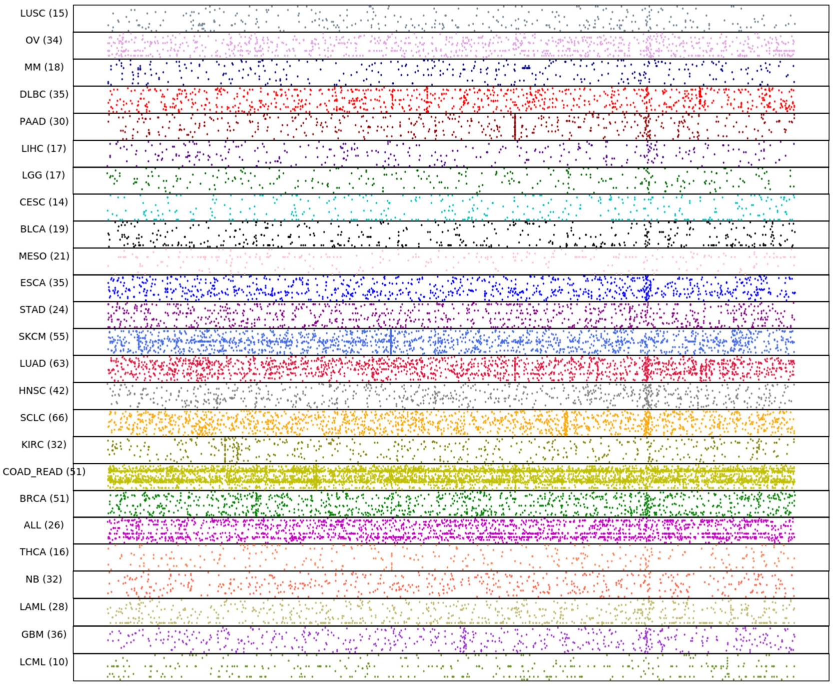

	NC	NG	NM	R^2
**NC**	1.00	0.77	0.56	-0.09
**NG**	0.77	1.00	0.79	-0.18
**NM**	0.56	0.79	1.00	-0.13
**R^2**	-0.09	-0.18	-0.13	1.00

NC: Number of cell lines per cancer type
NG: Total number of mutated genes per cancer type
NM: Total number of mutations per cancer type

A plot represents the mutational status across 28,328 positions for all cell lines over 25 cancer types. Cancer types and the number of relevant cell lines are indicated on the left of each row. The list of the caner types are sorted by R^2 values, with the highest R^2 value on the top. The R^2 values of individual cancer types are listed in the Supplementary Fig. S5. Each row represents a cell line, and individual dots indicate mutated base positions.

Supplementary Figure S5. Assessment of prediction accuracy of CDRscan™ for individual cancer types.

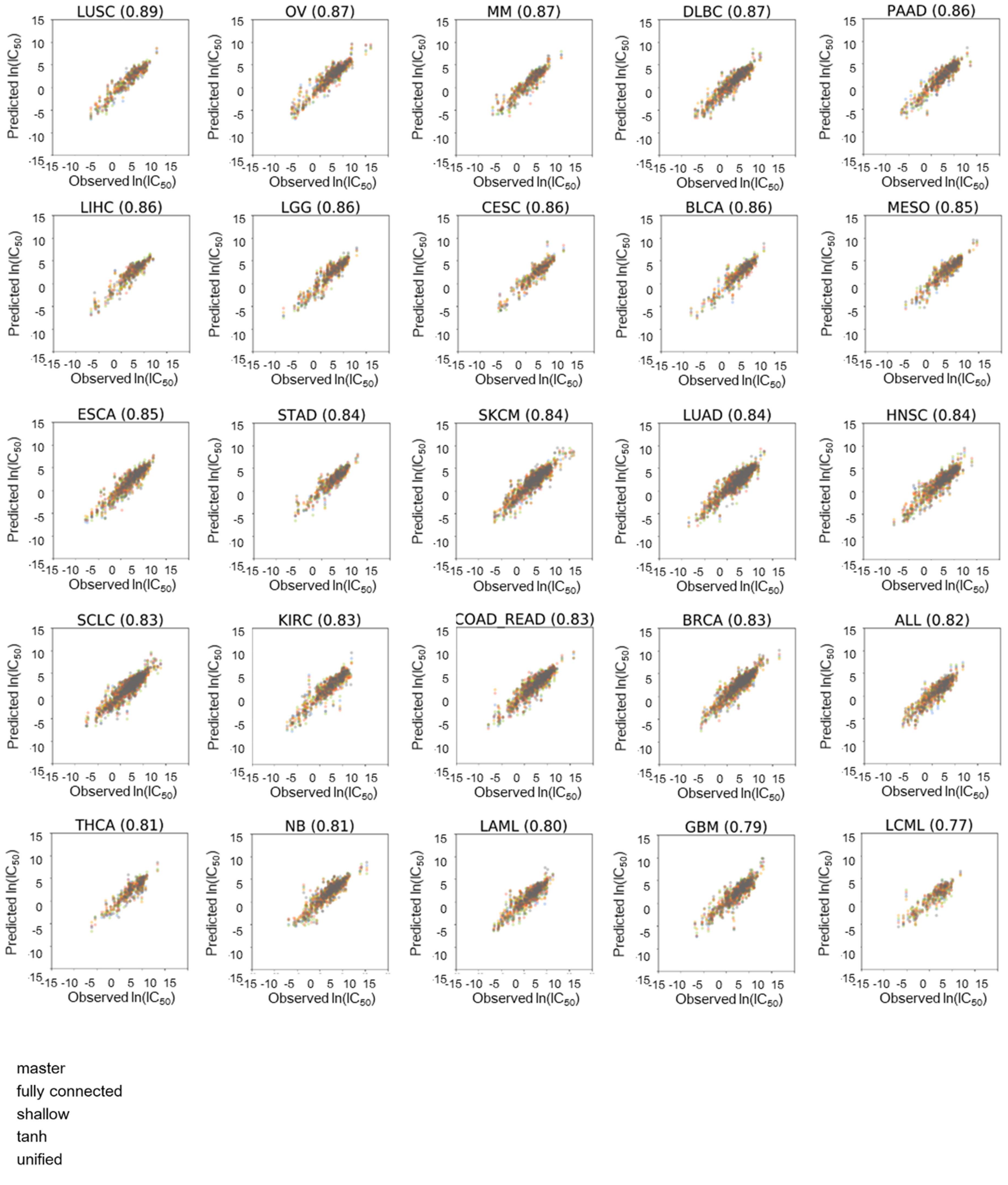

Each scatter plot shows a correlation between the observed and the CDRscan-predicted IC_{50} values in a natural log scale (in x- and y-axis, respectively) for individual caner types. For each cancer type, 5% of the instances that were not used for model training were used to validate five models of CDRscan. Scatter plots of the five models are superimposed on top of one another, generating one plot per cancer type. The cancer type and the mean R^2 values of the five models are indicated on the top of each plot, and the color keys are shown on the bottom of the figure.

Supplementary Figure S6. Cell line-centric correlation analysis.

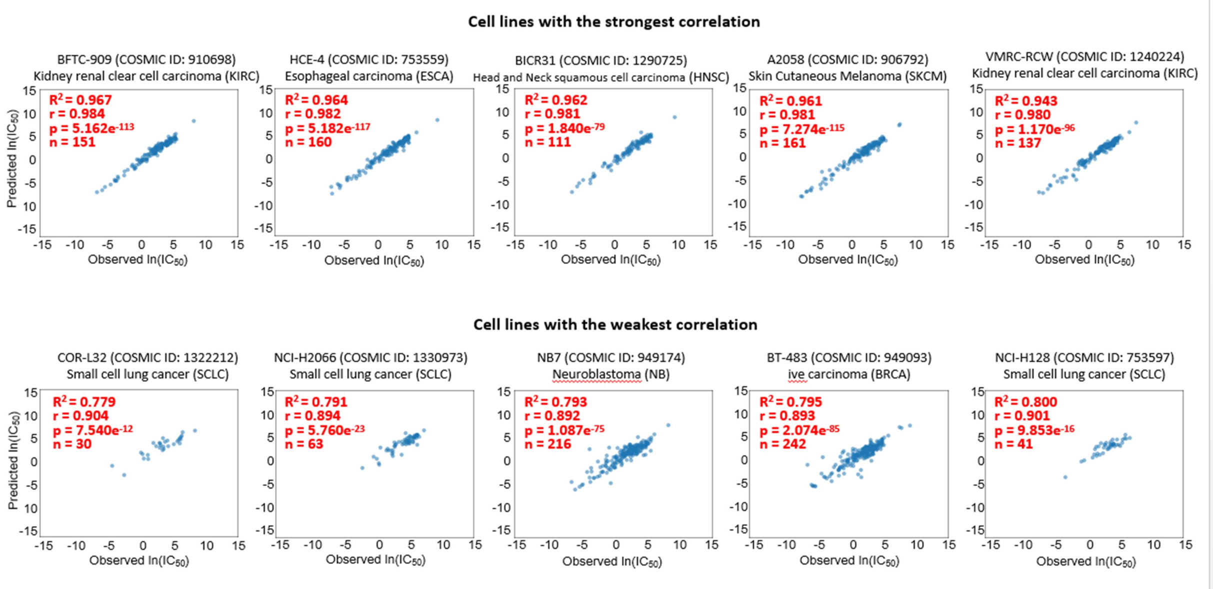

Scatter plots showing the degrees of correlation between the observed and the CDRscan-predicted $\ln(IC_{50})$ values for 10 cell lines, showing 5 top cell lines with the strongest (top) and 5 bottom ones with the lowest R^2 values (bottom). Each plot is labeled with the name of the cell line, the COSMIC ID, and corresponding cancer type. The R^2, Pearson correlation coefficient (r), p values, and instance counts are indicated at the upper left corner of each plot.

Supplementary Figure S7. Drug-centric correlation analysis.

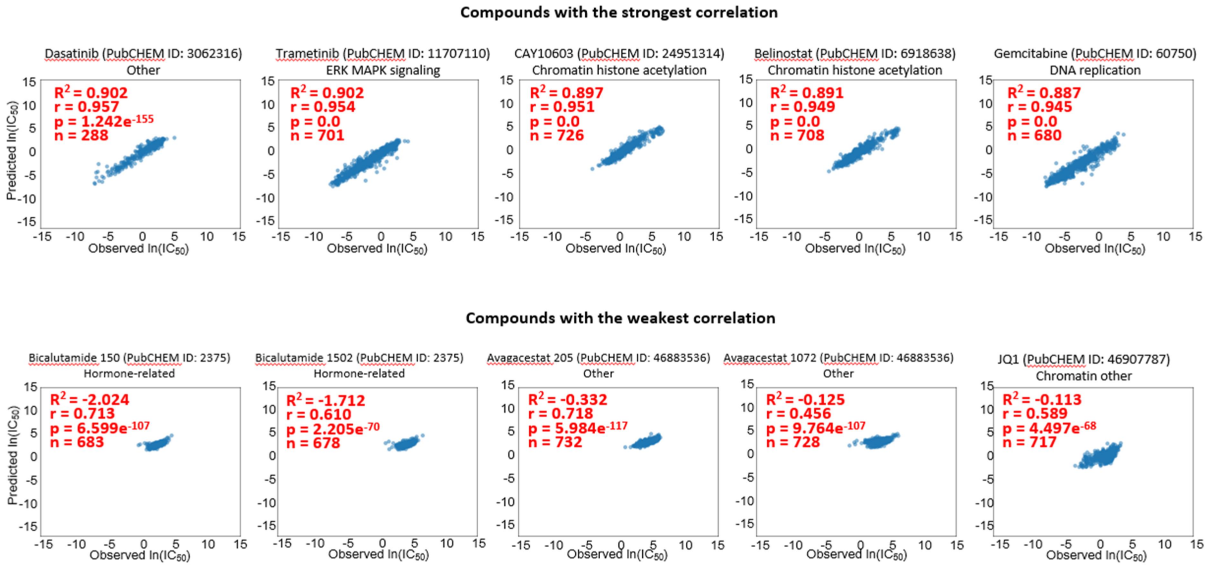

Scatter plots showing the degrees of correlation between the observed and the CDRscan-predicted ln(IC_{50}) values for 10 drugs, showing the 5 top drugs with strongest (top) and 5 bottom drugs with the lowest R^2 (bottom). Each drug is labeled with its name, PubChem ID, and its target pathway. The R^2, Pearson correlation coefficient (r), p values, and instance counts are indicated at the upper left corner of each plot.

Supplementary Figure S8. Five-fold cross validation of CDRscan.

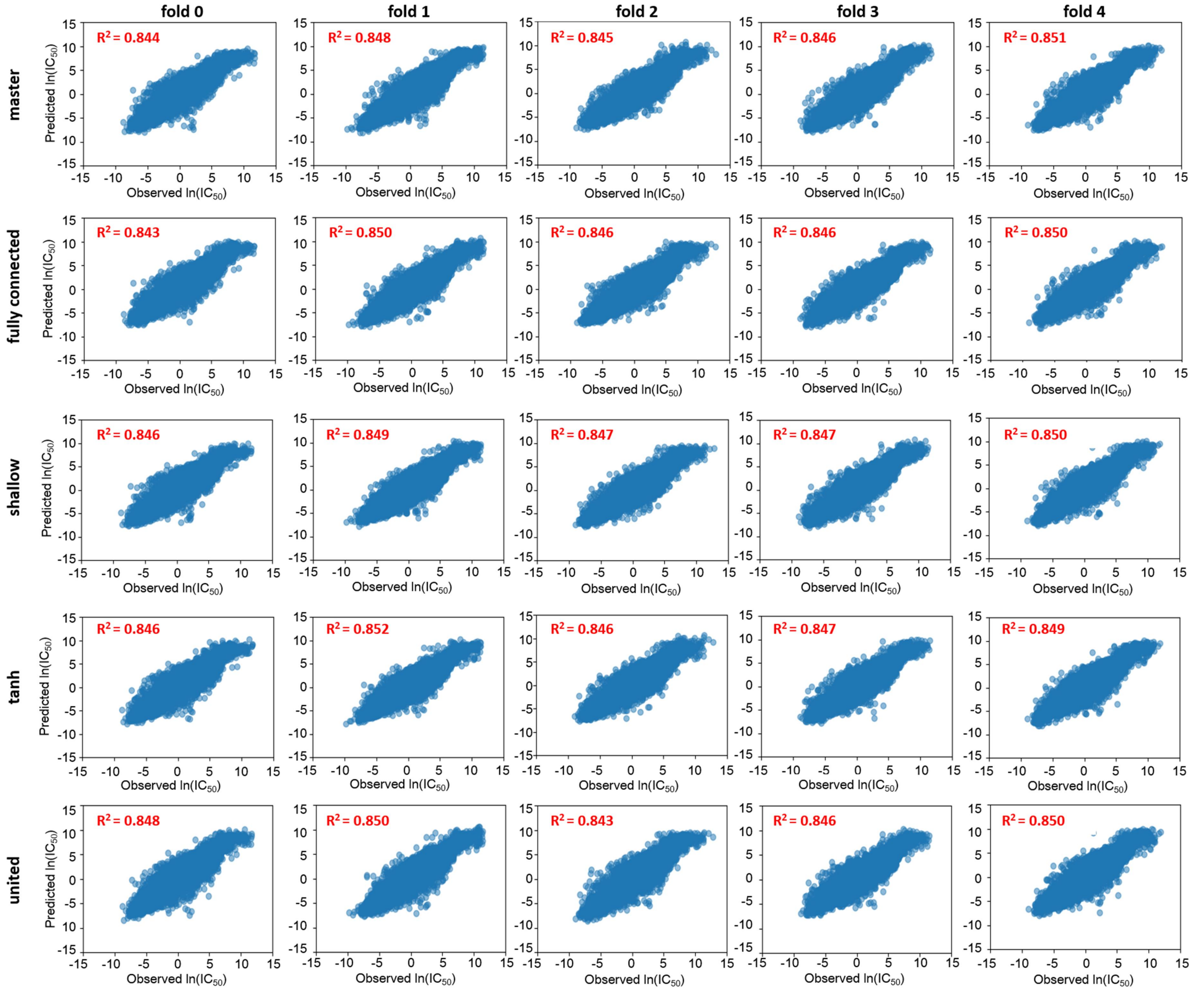

The performance of five independent models of CDRscan were evaluated by five-fold cross validation. The total data sets were randomly split into five equal sized subsamples. Each model was trained with data sets from four of the subsamples, leaving one subsample as a test set. The R^2 was then computed. This process was repeated four additional times, each time leaving different subgroup as a test set.

Continues on the next page

Continues on the next page

Supplementary Table S1. Architectures of five models of CDRscan.

Master

Layer	Filter	Pooling	Stride	Dense	Dropout	Activation Function	Output Shape	Param #
**Genomic profile**								
Convolution	(700, 50)	-	5	-	-	tanh	(5,526, 50)	35,050
Max pooling	-	5	-	-	-	-	(1,105, 50)	-
Convolution	(5, 30)	-	2	-	-	relu	(551, 30)	7,530
Max pooling	-	10	-	-	-	-	(55,30)	-
Flatten	-	-	-	-	-	-	1,650	-
Dense	-	-	-	100	-	relu	100	165,100
Dropout	-	-	-	-	0.1	-	100	-
**Chemical profile**								
Convolution	(200, 50)	-	3	-	-	tanh	(958, 50)	10,050
Max pooling	-	5	-	-	-	-	(191, 50)	-
Convolution	(50, 30)	-	5	-	-	relu	(29, 30)	75,030
Max pooling	-	10	-	-	-	-	(2, 30)	-
Flatten	-	-	-	-	-	-	60	-
Dense	-	-	-	100	-	relu	100	6,100
Dropout	-	-	-	-	0.1	-	100	-
**Merged**								
Concatenate	-	-	-	-	-	-	200	-
Dense	-	-	-	300	-	tanh	300	60,300
Dropout	-	-	-	-	0.1	-	300	-
Convolution	(150, 30)	-	1	-	-	relu	(151, 30)	4,530
Max pooling	-	2	-	-	-	-	(75, 30)	-
Convolution	(5, 10)	-	1	-	-	relu	(71, 10)	1,510
Max pooling	-	3	-	-	-	-	(23, 10)	-
Convolution	(5, 5)	-	1	-	-	relu	(19, 5)	255
Max pooling	-	3	-	-	-	-	(6, 5)	-
Dropout	-	-	-	-	0.1	-	(6, 5)	-
Flatten	-	-	-	-	-	-	30	-
Dropout	-	-	-	-	0.2	-	30	-
Dense	-	-	-	1	-	linear	1	31
Total								365,486

Continues on the next page

Fully connected

Layer	Filter	Pooling	Stride	Dense	Dropout	Activation Function	Output Shape	Param #
**Genomic profile**								
Convolution	(700, 50)	-	5	-	-	tanh	(5,526, 50)	35,050
Max pooling	-	5	-	-	-	-	(1,105, 50)	-
Convolution	(5, 30)	-	2	-	-	relu	(551, 30)	7,530
Max pooling	-	10	-	-	-	-	(55,30)	-
Flatten	-	-	-	-	-	-	1650	-
Dense	-	-	-	100	-	relu	100	165,100
Dropout	-	-	-	-	0.1	-	100	-
**Chemical profile**								
Convolution	(200, 50)	-	3	-	-	tanh	(958, 50)	10,050
Max pooling	-	5	-	-	-	-	(191, 50)	-
Convolution	(50, 30)	-	5	-	-	relu	(29, 30)	75,030
Max pooling	-	10	-	-	-	-	(2, 30)	-
Flatten	-	-	-	-	-	-	60	-
Dense	-	-	-	100	-	relu	100	6,100
Dropout	-	-	-	-	0.1	-	100	-
**Merged**								
Concatenate	-	-	-	-	-	-	200	-
Dense	-	-	-	300	-	tanh	300	60,300
Dropout	-	-	-	-	0.1	-	300	-
Convolution	(150, 30)	-	1	-	-	relu	(151, 30)	4,530
Max pooling	-	2	-	-	-	-	(75, 30)	-
Convolution	(5, 10)	-	1	-	-	relu	(71, 10)	1,510
Max pooling	-	3	-	-	-	-	(23, 10)	-
Convolution	(5, 5)	-	1	-	-	relu	(19, 5)	255
Max pooling	-	3	-	-	-	-	(6, 5)	-
Dropout	-	-	-	-	0.1	-	(6, 5)	-
Flatten	-	-	-	-	-	-	30	-
Dense	-	-	-	40	-	relu	40	1,240
Dropout	-	-	-	-	0.2	-	40	-
Dense	-	-	-	10	-	relu	10	410
Dropout	-	-	-	-	0.2	-	10	-
Dense	-	-	-	1	-	linear	1	11
Total								367,116

Continues on the next page

Shallow

Layer	Filter	Pooling	Stride	Dense	Dropout	Activation Function	Output Shape	Param #
**Genomic profile**								
Convolution	(700, 64)	-	5	-	-	tanh	(5,526, 64)	44,864
Max pooling	-	5	-	-	-	-	(1,105, 64)	-
Flatten	-	-	-	-	-	-	70,720	-
Dense	-	-	-	100	-	relu	100	7,072,100
Dropout	-	-	-	-	0.1	-	100	-
**Chemical profile**								
Convolution	(200, 64)	-	3	-	-	tanh	(958, 64)	12,864
Max pooling	-	5	-	-	-	-	(191, 64)	-
Flatten	-	-	-	-	-	-	12,224	-
Dense	-	-	-	100	-	relu	100	1,222,500
Dropout	-	-	-	-	0.1	-	100	-
**Merged**								
Concatenate	-	-	-	-	-	-	200	-
Dense	-	-	-	300	-	tanh	300	60,300
Dropout	-	-	-	-	0.1	-	300	-
Convolution	(150, 30)	-	1	-	-	relu	(151, 30)	4,530
Max pooling	-	2	-	-	-	-	(75, 30)	-
Convolution	(5, 10)	-	1	-	-	relu	(71, 10)	1,510
Max pooling	-	3	-	-	-	-	(23, 10)	-
Dropout	-	-	-	-	0.1	-	(23, 10)	-
Flatten	-	-	-	-	-	-	230	-
Dense	-	-	-	40	-	-	40	9,240
Dropout	-	-	-	-	0.2	-	40	-
Dense	-	-	-	1	-	linear	1	41
Total								8,427,949

Continues on the next page

Tanh

Layer	Filter	Pooling	Stride	Dense	Dropout	Activation Function	Output Shape	Param #
**Genomic profile**								
Batch Normalization	-	-	-	-	-	-	(28,328,1)	4
Convolution	(700, 50)	-	5	-	-	tanh	(5,526, 50)	35,050
Max pooling	-	5	-	-	-	-	(1,105, 50)	-
Convolution	(5, 30)	-	2	-	-	relu	(551, 30)	7,530
Max pooling	-	10	-	-	-	-	(55,30)	-
Flatten	-	-	-	-	-	-	1650	-
Dense	-	-	-	100	-	relu	100	165,100
Dropout	-	-	-	-	0.1	-	100	-
**Chemical profile**								
Batch Normalization							(3,072,1)	4
Convolution	(200, 50)	-	3	-	-	tanh	(958, 50)	10,050
Max pooling	-	5	-	-	-	-	(191, 50)	-
Convolution	(50, 30)	-	5	-	-	relu	(29, 30)	75,030
Max pooling	-	10	-	-	-	-	(2, 30)	-
Flatten	-	-	-	-	-	-	60	-
Dense	-	-	-	100	-	relu	100	6,100
Dropout	-	-	-	-	0.1	-	100	-
**Merged**								
Concatenate	-	-	-	-	-	-	200	-
Dense	-	-	-	300	-	tanh	300	60,300
Dropout	-	-	-	-	0.1	-	300	-
Convolution	(150, 30)	-	1	-	-	relu	(151, 30)	4,530
Max pooling	-	2	-	-	-	-	(75, 30)	-
Convolution	(5, 10)	-	1	-	-	relu	(71, 10)	1,510
Max pooling	-	3	-	-	-	-	(23, 10)	-
Convolution	(5, 5)	-	1	-	-	relu	(19, 5)	255
Max pooling	-	3	-	-	-	-	(6, 5)	-
Dropout	-	-	-	-	0.1	-	(6, 5)	-
Flatten	-	-	-	-	-	-	30	-
Dense	-	-	-	40	-	relu	40	1,240
Dropout	-	-	-	-	0.2	-	40	-
Dense	-	-	-	10	-	relu	10	410
Dropout	-	-	-	-	0.2	-	10	-
Dense	-	-	-	1	-	tanh	1	11
Tatal								367,124

Continues on the next page

Unified

Layer	Filter	Pooling	Stride	Dense	Dropout	Activation Function	Output Shape	Param #
Convolution	(700, 50)	-	5	-	-	tanh	(6,141, 50)	35,050
Max pooling	-	5	-	-	-	-	(1,228, 50)	-
Convolution	(5, 30)	-	2	-	-	relu	(612, 30)	7,530
Max pooling	-	10	-	-	-	-	(61,30)	-
Flatten	-	-	-	-	-	-	1830	-
Dense	-	-	-	100	-	relu	100	183,100
Dropout	-	-	-	-	0.1	-	100	-
Dense	-	-	-	300	-	tanh	300	30,300
Dropout	-	-	-	-	0.1	-	300	-
Convolution	(150, 30)	-	1	-	-	relu	(151, 30)	4,530
Max pooling	-	2	-	-	-	-	(75, 30)	-
Convolution	(5, 10)	-	1	-	-	relu	(71, 10)	1,510
Max pooling	-	3	-	-	-	-	(23, 10)	-
Convolution	(5, 5)	-	1	-	-	relu	(19, 5)	255
Max pooling	-	3	-	-	-	-	(6, 5)	-
Dropout	-	-	-	-	0.1	-	(6,5)	-
Flatten	-	-	-	-	-	-	30	-
Dropout	-	-	-	-	0.2	-	30	-
Dense	-	-	-	1	-	linear	1	31
Total								262,306

Continues on the next page

Supplementary Table S2. List of 787 COSMIC cell lines used for training and validation of CDRscan

No	COSMIC ID	Cell line name	Cancer Type
1	910944	ALL-PO	ALL
2	1295740	ALL-SIL	ALL
3	910687	ATN-1	ALL
4	906763	BE-13	ALL
5	905952	CCRF-CEM	ALL
6	1297446	DND-41	ALL
7	906877	GR-ST	ALL
8	949153	HAL-01	ALL
9	998184	Jurkat	ALL
10	907272	KARPAS-45	ALL
11	907277	KE-37	ALL
12	907787	LC4-1	ALL
13	907789	LOUCY	ALL
14	908146	MOLT-13	ALL
15	908147	MOLT-16	ALL
16	905958	MOLT-4	ALL
17	1330960	MY-M12	ALL
18	909251	P12-ICHIKAWA	ALL
19	909252	P30-OHK	ALL
20	909260	PF-382	ALL
21	909702	RPMI-8402	ALL
22	909703	RS4-11	ALL
23	1247871	SUP-B15	ALL
24	909743	SUP-T1	ALL
25	909762	TALL-1	ALL
26	946358	YT	ALL
27	687452	5637	BLCA
28	906798	639-V	BLCA
29	906797	647-V	BLCA
30	910926	BFTC-905	BLCA
31	1290730	CAL-29	BLCA
32	753552	DSH1	BLCA
33	907065	HT-1197	BLCA
34	907066	HT-1376	BLCA
35	753566	J82	BLCA
36	907312	KU-19-19	BLCA
37	753584	LB831-BLC	BLCA
38	909704	RT-112	BLCA
39	687455	RT4	BLCA
40	909749	SW1710	BLCA
41	687457	SW780	BLCA
42	724812	T-24	BLCA
43	687459	TCCSUP	BLCA
44	724838	UM-UC-3	BLCA
45	909780	VM-CUB-1	BLCA
46	910704	AU565	BRCA
47	906801	BT-20	BRCA
48	946359	BT-474	BRCA
49	949093	BT-483	BRCA
50	905951	BT-549	BRCA
51	906826	CAL-120	BRCA
52	924106	CAL-148	BRCA
53	910927	CAL-51	BRCA
54	910852	CAL-85-1	BRCA
55	946382	CAMA-1	BRCA
56	906812	COLO-824	BRCA
57	906844	DU-4475	BRCA
58	906851	EFM-19	BRCA
59	1290798	EFM-192A	BRCA
60	906862	EVSA-T	BRCA
61	749710	HCC1143	BRCA
62	749711	HCC1187	BRCA
63	749712	HCC1395	BRCA
64	907045	HCC1419	BRCA
65	1290905	HCC1428	BRCA
66	1303900	HCC1500	BRCA
67	907046	HCC1569	BRCA
68	749713	HCC1599	BRCA
69	749714	HCC1937	BRCA
70	749709	HCC1954	BRCA
71	1290906	HCC202	BRCA
72	749715	HCC2157	BRCA
73	749716	HCC2218	BRCA
74	749717	HCC38	BRCA
75	907048	HCC70	BRCA
76	1290922	HDQ-P1	BRCA
77	905957	Hs-578-T	BRCA
78	1298157	JIMT-1	BRCA
79	905946	MCF7	BRCA
80	925338	MDA-MB-157	BRCA
81	908120	MDA-MB-175-VII	BRCA
82	905960	MDA-MB-231	BRCA
83	1330941	MDA-MB-330	BRCA
84	908121	MDA-MB-361	BRCA
85	924240	MDA-MB-415	BRCA
86	1240172	MDA-MB-436	BRCA
87	908122	MDA-MB-453	BRCA
88	908123	MDA-MB-468	BRCA
89	910948	MFM-223	BRCA
90	908151	MRK-nu-1	BRCA
91	909256	OCUB-M	BRCA
92	905945	T47D	BRCA
93	910910	UACC-812	BRCA
94	909778	UACC-893	BRCA
95	1303911	YMB-1-E	BRCA
96	909907	ZR-75-30	BRCA
97	687505	C-33-A	CESC
98	687506	C-4-I	CESC
99	924107	CAL-39	CESC
100	906824	Ca-Ski	CESC
101	906843	DoTc2-4510	CESC
102	1298134	HeLa	CESC
103	907068	HT-3	CESC
104	687514	ME-180	CESC
105	1240179	MS751	CESC
106	949154	OMC-1	CESC
107	930297	SiHa	CESC
108	1240212	SISO	CESC
109	930298	SKG-IIIa	CESC
110	724839	SW756	CESC
111	910700	C2BBe1	COAD_READ
112	924108	CaR-1	COAD_READ
113	1240123	CCK-81	COAD_READ
114	1290769	CL-11	COAD_READ
115	1290771	CL-34	COAD_READ
116	1240124	CL-40	COAD_READ
117	905961	COLO-205	COAD_READ
118	910569	COLO-320-HSR	COAD_READ
119	910689	COLO-678	COAD_READ
120	910554	CW-2	COAD_READ
121	1789883	DIFI	COAD_READ
122	907291	GP5d	COAD_READ
123	905971	HCC2998	COAD_READ
124	1290907	HCC-56	COAD_READ
125	905936	HCT-116	COAD_READ
126	905937	HCT-15	COAD_READ
127	907289	HT-115	COAD_READ
128	905939	HT-29	COAD_READ
129	907287	HT55	COAD_READ
130	905989	KM12	COAD_READ
131	907790	LoVo	COAD_READ
132	917486	LS-1034	COAD_READ
133	907792	LS-123	COAD_READ
134	998189	LS-180	COAD_READ
135	907794	LS-411N	COAD_READ
136	907795	LS-513	COAD_READ
137	1240173	MDST8	COAD_READ
138	908442	NCI-H508	COAD_READ
139	908482	NCI-H630	COAD_READ
140	908458	NCI-H716	COAD_READ
141	908457	NCI-H747	COAD_READ
142	909263	RCM-1	COAD_READ
143	909698	RKO	COAD_READ
144	909718	SK-CO-1	COAD_READ

Continues on the next page

No	COSMIC ID	Cell line name	Cancer Type
145	1659823	SNU-1040	COAD_READ
146	1659928	SNU-175	COAD_READ
147	1659929	SNU-283	COAD_READ
148	1660034	SNU-407	COAD_READ
149	1660035	SNU-61	COAD_READ
150	1660036	SNU-81	COAD_READ
151	910905	SNU-C1	COAD_READ
152	909740	SNU-C2B	COAD_READ
153	1674021	SNU-C5	COAD_READ
154	909746	SW1116	COAD_READ
155	909747	SW1417	COAD_READ
156	909748	SW1463	COAD_READ
157	909751	SW48	COAD_READ
158	905962	SW620	COAD_READ
159	909755	SW837	COAD_READ
160	909757	SW948	COAD_READ
161	909761	T84	COAD_READ
162	910935	A3-KAW	DLBC
163	910934	A4-Fuk	DLBC
164	910919	BC-1	DLBC
165	910918	BC-3	DLBC
166	906807	CRO-AP2	DLBC
167	949088	CTB-1	DLBC
168	906832	DB	DLBC
169	906842	DOHH-2	DLBC
170	1297449	Farage	DLBC
171	1303897	GRANTA-519	DLBC
172	907063	HT	DLBC
173	1327765	JEKO-1	DLBC
174	1327768	JM1	DLBC
175	1327769	JSC-1	DLBC
176	1327773	KARPAS-1106P	DLBC
177	907274	KARPAS-422	DLBC
178	907799	MC116	DLBC
179	1330982	NU-DUL-1	DLBC
180	1330984	OCI-LY-19	DLBC
181	1659819	OCI-LY7	DLBC
182	1330995	RC-K8	DLBC
183	910861	RL	DLBC
184	1331030	SC-1	DLBC
185	910930	SCC-3	DLBC
186	1331032	SLVL	DLBC
187	1331033	SU-DHL-10	DLBC
188	1331034	SU-DHL-16	DLBC
189	1331035	SU-DHL-4	DLBC
190	1331036	SU-DHL-5	DLBC
191	1331037	SU-DHL-6	DLBC
192	1331038	SU-DHL-8	DLBC
193	1331045	TK	DLBC
194	909773	TUR	DLBC
195	1331048	VAL	DLBC
196	1331050	WSU-DLCL2	DLBC
197	906817	COLO-680N	ESCA
198	753555	EC-GI-10	ESCA
199	1503366	ESO26	ESCA
200	1503367	ESO51	ESCA
201	1503361	FLO-1	ESCA
202	753559	HCE-4	ESCA
203	1503368	KYAE-1	ESCA
204	753573	KYSE-140	ESCA
205	907317	KYSE-150	ESCA
206	907318	KYSE-180	ESCA
207	1240167	KYSE-220	ESCA
208	907319	KYSE-270	ESCA
209	753574	KYSE-410	ESCA
210	907320	KYSE-450	ESCA
211	1298222	KYSE-50	ESCA
212	907321	KYSE-510	ESCA
213	753575	KYSE-520	ESCA
214	753576	KYSE-70	ESCA
215	1503363	OACM5-1	ESCA
216	1503362	OACp4C	ESCA
217	910079	OE19	ESCA
218	1298359	OE21	ESCA
219	910549	OE33	ESCA
220	1503365	SK-GT-4	ESCA
221	753621	TE-1	ESCA
222	753622	TE-10	ESCA
223	946354	TE-11	ESCA
224	946356	TE-12	ESCA
225	753614	TE-15	ESCA
226	1503371	TE-4	ESCA
227	735784	TE-5	ESCA
228	946355	TE-6	ESCA
229	753623	TE-8	ESCA
230	946353	TE-9	ESCA
231	1299064	T-T	ESCA
232	687561	42-MG-BA	GBM
233	687562	8-MG-BA	GBM
234	687563	A172	GBM
235	910933	AM-38	GBM
236	906746	Becker	GBM
237	910943	CAS-1	GBM
238	906823	CCF-STTG1	GBM
239	946366	D-245MG	GBM
240	946367	D-247MG	GBM
241	946368	D-263MG	GBM
242	946370	D-392MG	GBM
243	946372	D-423MG	GBM
244	753549	D-542MG	GBM
245	946377	D-566MG	GBM
246	906835	DBTRG-05MG	GBM
247	906839	DK-MG	GBM
248	687568	GB-1	GBM
249	906873	GMS-10	GBM
250	907313	KS-1	GBM
251	1240168	LN-18	GBM
252	1240169	LN-229	GBM
253	910694	LN-405	GBM
254	1240170	LNZTA3WT4	GBM
255	949094	M059J	GBM
256	909712	SF126	GBM
257	905986	SF268	GBM
258	905985	SF295	GBM
259	909729	SK-MG-1	GBM
260	905982	SNB75	GBM
261	909745	SW1088	GBM
262	687586	T98G	GBM
263	687588	U-118-MG	GBM
264	905983	U251	GBM
265	687590	U-87-MG	GBM
266	909905	YH-13	GBM
267	687592	YKG-1	GBM
268	753531	BB30-HNC	HNSC
269	753532	BB49-HNC	HNSC
270	753535	BHY	HNSC
271	1290724	BICR10	HNSC
272	1240121	BICR22	HNSC
273	1290725	BICR31	HNSC
274	1240122	BICR78	HNSC
275	753538	Ca9-22	HNSC
276	910916	CAL-27	HNSC
277	753541	CAL-33	HNSC
278	906837	Detroit562	HNSC
279	910936	DOK	HNSC
280	906863	FADU	HNSC
281	907059	HN	HNSC
282	924111	HO-1-N-1	HNSC
283	753561	HO-1-u-1	HNSC
284	753562	HSC-2	HNSC
285	907061	HSC-3	HNSC
286	907062	HSC-4	HNSC
287	1240161	JHU-011	HNSC
288	1240162	JHU-022	HNSC
289	1298156	JHU-029	HNSC
290	1298215	KON	HNSC
291	753570	KOSC-2	HNSC

Continues on the next page

No	COSMIC ID	Cell line name	Cancer Type
292	753583	LB771-HNC	HNSC
293	1298362	OSC-19	HNSC
294	1240196	OSC-20	HNSC
295	1240204	PCI-15A	HNSC
296	1298529	PCI-30	HNSC
297	1240205	PCI-38	HNSC
298	1298531	PCI-4B	HNSC
299	1240206	PCI-6A	HNSC
300	1240207	PE/CA-PJ15	HNSC
301	909700	RPMI-2650	HNSC
302	909708	SAS	HNSC
303	1299050	SAT	HNSC
304	910911	SCC-15	HNSC
305	910701	SCC-25	HNSC
306	910904	SCC-4	HNSC
307	909709	SCC-9	HNSC
308	1299052	SCC90	HNSC
309	1299059	SKN-3	HNSC
310	910922	769-P	KIRC
311	905947	786-0	KIRC
312	905948	A498	KIRC
313	910920	A704	KIRC
314	905950	ACHN	KIRC
315	753533	BB65-RCC	KIRC
316	910698	BFTC-909	KIRC
317	905963	CAKI-1	KIRC
318	910952	CAL-54	KIRC
319	753558	HA7-RCC	KIRC
320	1298168	KMRC-1	KIRC
321	1298169	KMRC-20	KIRC
322	753577	LB1047-RCC	KIRC
323	753578	LB2241-RCC	KIRC
324	753585	LB996-RCC	KIRC
325	1509073	NCC010	KIRC
326	1509074	NCC021	KIRC
327	909250	OS-RC-2	KIRC
328	909974	RCC10RGB	KIRC
329	1524418	RCC-AB	KIRC
330	1524417	RCC-ER	KIRC
331	1524414	RCC-FG2	KIRC
332	1524415	RCC-JF	KIRC
333	1524416	RCC-JW	KIRC
334	1524419	RCC-MF	KIRC
335	905978	RXF393	KIRC
336	905979	SN12C	KIRC
337	1240220	SW156	KIRC
338	905980	TK10	KIRC
339	905981	U031	KIRC
340	1240224	VMRC-RCW	KIRC
341	909781	VMRC-RCZ	KIRC
342	910688	CESS	LAML
343	910566	CMK	LAML
344	753548	CTV-1	LAML
345	906870	GDM-1	LAML
346	907053	HEL	LAML
347	905938	HL-60	LAML
348	907275	KASUMI-1	LAML
349	907278	KG-1	LAML
350	907280	KMOE-2	LAML
351	1330932	KO52	LAML
352	907300	KY821	LAML
353	1330942	ME-1	LAML
354	908141	ML-2	LAML
355	1330947	MOLM-13	LAML
356	1330948	MOLM-16	LAML
357	908148	MONO-MAC-6	LAML
358	1323913	NB-4	LAML
359	908448	NKM-1	LAML
360	908451	NOMO-1	LAML
361	910947	OCI-AML2	LAML
362	1290455	OCI-AML3	LAML
363	1330983	OCI-AML5	LAML
364	1330985	OCI-M1	LAML
365	909253	P31-FUJ	LAML
366	1330991	PL-21	LAML
367	910545	QIMR-WIL	LAML
368	909715	SIG-M5	LAML
369	909771	THP-1	LAML
370	910710	BV-173	LCML
371	910951	CML-T1	LCML
372	906855	EM-2	LCML
373	1327771	JURL-MK1	LCML
374	905940	K-562	LCML
375	1330931	KCL-22	LCML
376	907311	KU812	LCML
377	907783	LAMA-84	LCML
378	908126	MEG-01	LCML
379	910544	RPMI-8866	LCML
380	946369	D-336MG	LGG
381	946373	D-502MG	LGG
382	906868	GAMG	LGG
383	906871	GI-1	LGG
384	907042	H4	LGG
385	1240150	Hs683	LGG
386	907271	KALS-1	LGG
387	907279	KINGS-1	LGG
388	907282	KNS-42	LGG
389	924188	KNS-81-FD	LGG
390	908144	MOG-G-CCM	LGG
391	908145	MOG-G-UVW	LGG
392	908449	NMC-G1	LGG
393	908452	no-10	LGG
394	908450	no-11	LGG
395	905984	SF539	LGG
396	909750	SW1783	LGG
397	910850	C3A	LIHC
398	1240147	Hep 3B2_1-7	LIHC
399	907057	HLE	LIHC
400	1298146	huH-1	LIHC
401	907071	HuH-7	LIHC
402	1298151	JHH-1	LIHC
403	1240157	JHH-2	LIHC
404	1240158	JHH-4	LIHC
405	1240159	JHH-6	LIHC
406	1240160	JHH-7	LIHC
407	909719	SK-HEP-1	LIHC
408	1240216	SNU-182	LIHC
409	909736	SNU-387	LIHC
410	1240217	SNU-398	LIHC
411	909737	SNU-423	LIHC
412	909738	SNU-449	LIHC
413	909739	SNU-475	LIHC
414	1287381	201T	LUAD
415	905949	A549	LUAD
416	906791	ABC-1	LUAD
417	687777	Calu-3	LUAD
418	724859	Calu-6	LUAD
419	906805	COR-L105	LUAD
420	905970	EKVX	LUAD
421	1503369	EMC-BAC-1	LUAD
422	1503370	EMC-BAC-2	LUAD
423	1247873	H3255	LUAD
424	1240145	HCC-44	LUAD
425	1290908	HCC-78	LUAD
426	1240146	HCC-827	LUAD
427	905972	HOP-62	LUAD
428	907786	LC-2-ad	LUAD
429	753592	LXF-289	LUAD
430	724866	NCI-H1355	LUAD
431	1298347	NCI-H1435	LUAD
432	687794	NCI-H1437	LUAD
433	753600	NCI-H1563	LUAD
434	1298348	NCI-H1568	LUAD
435	908472	NCI-H1573	LUAD

Continues on the next page

No	COSMIC ID	Cell line name	Cancer Type
436	687798	NCI-H1623	LUAD
437	687799	NCI-H1648	LUAD
438	687800	NCI-H1650	LUAD
439	910900	NCI-H1651	LUAD
440	908473	NCI-H1666	LUAD
441	687802	NCI-H1693	LUAD
442	722058	NCI-H1734	LUAD
443	908475	NCI-H1755	LUAD
444	1298350	NCI-H1781	LUAD
445	724868	NCI-H1792	LUAD
446	908463	NCI-H1793	LUAD
447	687807	NCI-H1838	LUAD
448	1240185	NCI-H1944	LUAD
449	924244	NCI-H1975	LUAD
450	908476	NCI-H1993	LUAD
451	724873	NCI-H2009	LUAD
452	1240187	NCI-H2023	LUAD
453	722045	NCI-H2030	LUAD
454	687812	NCI-H2085	LUAD
455	724834	NCI-H2087	LUAD
456	722046	NCI-H2122	LUAD
457	687816	NCI-H2228	LUAD
458	724874	NCI-H2291	LUAD
459	905942	NCI-H23	LUAD
460	687819	NCI-H2342	LUAD
461	687820	NCI-H2347	LUAD
462	687821	NCI-H2405	LUAD
463	1240190	NCI-H3122	LUAD
464	905967	NCI-H322M	LUAD
465	908465	NCI-H358	LUAD
466	908460	NCI-H441	LUAD
467	905944	NCI-H522	LUAD
468	722066	NCI-H650	LUAD
469	910399	NCI-H838	LUAD
470	753608	PC-14	LUAD
471	1240202	PC-3 [JPC-3]	LUAD
472	1298537	RERF-LC-KJ	LUAD
473	910931	RERF-LC-MS	LUAD
474	909721	SK-LU-1	LUAD
475	724878	SW1573	LUAD
476	713869	VMRC-LCD	LUAD
477	753554	EBC-1	LUSC
478	753556	EPLC-272H	LUSC
479	1240142	HARA	LUSC
480	1240143	HCC-15	LUSC
481	753569	KNS-62	LUSC
482	1298223	LC-1-sq	LUSC
483	687787	LK-2	LUSC
484	1298226	LOU-NH91	LUSC
485	1240183	NCI-H1869	LUSC
486	687815	NCI-H2170	LUSC
487	905941	NCI-H226	LUSC
488	908443	NCI-H520	LUSC
489	1298538	RERF-LC-Sq1	LUSC
490	909728	SK-MES-1	LUSC
491	724879	SW900	LUSC
492	1290808	H2369	MESO
493	1290809	H2373	MESO
494	1290810	H2461	MESO
495	1240131	H2591	MESO
496	1240132	H2595	MESO
497	1290812	H2722	MESO
498	1240134	H2731	MESO
499	1290813	H2795	MESO
500	1240135	H2803	MESO
501	1240136	H2804	MESO
502	1240137	H2810	MESO
503	1290814	H2818	MESO
504	1240138	H2869	MESO
505	1240139	H290	MESO
506	1240141	H513	MESO
507	907173	IST-MES1	MESO
508	908150	MPP-89	MESO
509	908152	MSTO-211H	MESO
510	688058	NCI-H2052	MESO
511	908462	NCI-H2452	MESO
512	908470	NCI-H28	MESO
513	1295741	AMO-1	MM
514	906765	ARH-77	MM
515	1297447	EJM	MM
516	753563	IM-9	MM
517	1327766	JJN-3	MM
518	1327775	KARPAS-620	MM
519	1659817	KMS-11	MM
520	907281	KMS-12-BM	MM
521	924239	L-363	MM
522	907791	LP-1	MM
523	683665	MC-CAR	MM
524	1659818	MM1S	MM
525	1330950	MOLP-8	MM
526	724825	NCI-H929	MM
527	909249	OPM-2	MM
528	905964	RPMI-8226	MM
529	753612	SK-MM-2	MM
530	753615	U-266	MM
531	753616	BE2-M17	NB
532	910567	CHP-126	NB
533	910941	CHP-134	NB
534	906820	CHP-212	NB
535	906872	GI-ME-N	NB
536	906875	GOTO	NB
537	907170	IMR-5	NB
538	753618	KELLY	NB
539	924189	KP-N-RT-BM-1	NB
540	907314	KP-N-YN	NB
541	946363	KP-N-YS	NB
542	949170	LAN-6	NB
543	908118	MC-IXC	NB
544	908135	MHH-NB-11	NB
545	949179	NB1	NB
546	949171	NB10	NB
547	949172	NB12	NB
548	949177	NB13	NB
549	949178	NB14	NB
550	949175	NB17	NB
551	949176	NB5	NB
552	949173	NB6	NB
553	908440	NB69	NB
554	949174	NB7	NB
555	1240181	NB(TU)1-10	NB
556	908447	NH-12	NB
557	753620	SIMA	NB
558	724828	SK-N-AS	NB
559	688086	SK-N-DZ	NB
560	688087	SK-N-FI	NB
561	717431	SK-N-SH	NB
562	910780	TGW	NB
563	906825	Caov-3	OV
564	949090	Caov-4	OV
565	1479987	DOV13	OV
566	911905	EFO-21	OV
567	1240129	FU-OV-1	OV
568	1479988	Hey	OV
569	905968	IGROV-1	OV
570	1479995	JHOS-2	OV
571	1480358	JHOS-3	OV
572	1480359	JHOS-4	OV
573	946360	OAW-28	OV
574	910548	OAW-42	OV
575	909257	OC-314	OV
576	1480361	OV-17R	OV
577	1480362	OV-56	OV
578	1480360	OV-7	OV
579	1240197	OV-90	OV

Continues on the next page

No	COSMIC ID	Cell line name	Cancer Type
580	1480364	OVCA420	OV
581	1480367	OVCA433	OV
582	905933	OVCAR-3	OV
583	905990	OVCAR-4	OV
584	905969	OVCAR-5	OV
585	905991	OVCAR-8	OV
586	1240198	OVISE	OV
587	1480371	OVK-18	OV
588	1240199	OVKATE	OV
589	1240200	OVMIU	OV
590	1298365	OVTOKO	OV
591	1480372	PEO1	OV
592	905959	SK-OV-3	OV
593	909753	SW626	OV
594	1299070	TOV-112D	OV
595	1240222	TOV-21G	OV
596	1480374	UWB1.289	OV
597	910702	AsPC-1	PAAD
598	906693	BxPC-3	PAAD
599	753624	CAPAN-1	PAAD
600	910915	Capan-2	PAAD
601	906821	CFPAC-1	PAAD
602	1290797	DAN-G	PAAD
603	1298136	HPAC	PAAD
604	724869	HPAF-II	PAAD
605	1298141	Hs766T	PAAD
606	907285	HuP-T3	PAAD
607	907286	HuP-T4	PAAD
608	1298216	KP-1N	PAAD
609	1298218	KP-2	PAAD
610	753572	KP-4	PAAD
611	724870	MIA-PaCa-2	PAAD
612	753595	MZ1-PC	PAAD
613	1298475	PANC-02-03	PAAD
614	925346	PANC-03-27	PAAD
615	1298476	PANC-04-03	PAAD
616	925347	PANC-08-13	PAAD
617	925348	PANC-10-05	PAAD
618	1298526	PA-TU-8902	PAAD
619	1240201	PA-TU-8988T	PAAD
620	1240208	PL18	PAAD
621	1298533	PL4	PAAD
622	910546	PSN1	PAAD
623	1240218	SU8686	PAAD
624	1240219	SUIT-2	PAAD
625	910907	SW1990	PAAD
626	909904	YAPC	PAAD
627	910692	COLO-668	SCLC
628	910937	COR-L279	SCLC
629	1297438	COR-L303	SCLC
630	687980	COR-L311	SCLC
631	1322212	COR-L32	SCLC
632	906808	COR-L88	SCLC
633	1297439	COR-L95	SCLC
634	753547	CPC-N	SCLC
635	687983	DMS-114	SCLC
636	687985	DMS-273	SCLC
637	907295	DMS-53	SCLC
638	753551	DMS-79	SCLC
639	1303901	HCC-33	SCLC
640	753564	IST-SL1	SCLC
641	753565	IST-SL2	SCLC
642	753582	LB647-SCLC	SCLC
643	753588	LU-134-A	SCLC
644	713899	LU-135	SCLC
645	713878	LU-139	SCLC
646	753589	LU-165	SCLC
647	753594	MS-1	SCLC
648	687995	NCI-H1048	SCLC
649	687997	NCI-H1092	SCLC
650	908468	NCI-H1105	SCLC
651	753597	NCI-H128	SCLC
652	753599	NCI-H1304	SCLC
653	1330964	NCI-H1341	SCLC
654	688001	NCI-H1417	SCLC
655	908469	NCI-H1436	SCLC
656	910899	NCI-H146	SCLC
657	1298349	NCI-H1688	SCLC
658	688006	NCI-H1694	SCLC
659	1240182	NCI-H1836	SCLC
660	688007	NCI-H187	SCLC
661	1330972	NCI-H1876	SCLC
662	1240186	NCI-H196	SCLC
663	688010	NCI-H1963	SCLC
664	688011	NCI-H2029	SCLC
665	1330973	NCI-H2066	SCLC
666	908480	NCI-H2081	SCLC
667	688013	NCI-H209	SCLC
668	1240189	NCI-H211	SCLC
669	688014	NCI-H2141	SCLC
670	688015	NCI-H2171	SCLC
671	908481	NCI-H2196	SCLC
672	688018	NCI-H2227	SCLC
673	924241	NCI-H250	SCLC
674	688021	NCI-H345	SCLC
675	688022	NCI-H378	SCLC
676	688023	NCI-H446	SCLC
677	753605	NCI-H510A	SCLC
678	908483	NCI-H524	SCLC
679	688025	NCI-H526	SCLC
680	688026	NCI-H64	SCLC
681	688027	NCI-H69	SCLC
682	1331055	NCI-H740	SCLC
683	909194	NCI-H748	SCLC
684	688031	NCI-H82	SCLC
685	1240192	NCI-H841	SCLC
686	1240193	NCI-H847	SCLC
687	930081	RERF-LC-FM	SCLC
688	713885	SBC-1	SCLC
689	753610	SBC-3	SCLC
690	713880	SBC-5	SCLC
691	724872	SHP-77	SCLC
692	1299062	SW1271	SCLC
693	1287706	451Lu	SKCM
694	910921	A101D	SKCM
695	906792	A2058	SKCM
696	906793	A375	SKCM
697	906830	C32	SKCM
698	910853	CHL-1	SKCM
699	906818	COLO-679	SKCM
700	1240125	COLO-783	SKCM
701	906814	COLO-792	SKCM
702	906813	COLO-800	SKCM
703	687448	COLO-829	SKCM
704	753545	CP50-MEL-B	SKCM
705	753546	CP66-MEL	SKCM
706	949092	CP67-MEL	SKCM
707	906865	G-361	SKCM
708	910932	GAK	SKCM
709	1240130	G-MEL	SKCM
710	907058	HMV-II	SKCM
711	1298144	Hs939-T	SKCM
712	1298145	Hs940-T	SKCM
713	907067	HT-144	SKCM
714	907169	IGR-1	SKCM
715	1240153	IGR-37	SKCM
716	907171	IPC-298	SKCM
717	907172	IST-MEL1	SKCM
718	1298160	K2	SKCM
719	753579	LB2518-MEL	SKCM
720	753581	LB373-MEL-D	SKCM
721	905974	LOXIMVI	SKCM
722	905975	M14	SKCM
723	908124	MEL-HO	SKCM
724	908125	MEL-JUSO	SKCM
725	908128	Mewo	SKCM
726	925339	MMAC-SF	SKCM

Continues on the next page

No	COSMIC ID	Cell line name	Cancer Type
727	971777	MZ2-MEL	SKCM
728	753596	MZ7-mel	SKCM
729	910903	RPMI-7951	SKCM
730	909706	RVH-421	SKCM
731	909713	SH-4	SKCM
732	909723	SK-MEL-1	SKCM
733	905955	SK-MEL-2	SKCM
734	909725	SK-MEL-24	SKCM
735	905954	SK-MEL-28	SKCM
736	909724	SK-MEL-3	SKCM
737	909726	SK-MEL-30	SKCM
738	909727	SK-MEL-31	SKCM
739	905956	SK-MEL-5	SKCM
740	905977	UACC-257	SKCM
741	905976	UACC-62	SKCM
742	930301	VMRC-MELG	SKCM
743	909784	WM-115	SKCM
744	1299078	WM1552C	SKCM
745	1240226	WM278	SKCM
746	1299080	WM35	SKCM
747	1299081	WM793B	SKCM
748	910924	23132-87	STAD
749	906790	AGS	STAD
750	1290806	FU97	STAD
751	906869	GCIY	STAD
752	907055	HGC-27	STAD
753	1240151	Hs746T	STAD
754	1322224	HSC-39	STAD
755	1240155	IM-95	STAD
756	907276	KATOIII	STAD
757	908139	MKN28	STAD
758	925340	MKN45	STAD
759	924250	MKN7	STAD
760	908461	NCI-N87	STAD
761	908444	NCI-SNU-1	STAD
762	908446	NCI-SNU-16	STAD
763	908445	NCI-SNU-5	STAD
764	908455	NUGC-3	STAD
765	1298357	NUGC-4	STAD
766	1298358	OCUM-1	STAD
767	1240209	RERF-GC-1B	STAD
768	909697	RF-48	STAD
769	1503364	SK-GT-2	STAD
770	909770	TGBC11TKB	STAD
771	1299069	TMK-1	STAD
772	906795	8305C	THCA
773	924102	8505C	THCA
774	1290722	ASH-3	THCA
775	924104	BCPAP	THCA
776	906696	BHT-101	THCA
777	906828	CAL-62	THCA
778	910568	CGTH-W-1	THCA
779	906864	FTC-133	THCA
780	924151	HTC-C3	THCA
781	1240154	IHH-4	THCA
782	924238	K5	THCA
783	1298167	KMH-2	THCA
784	1240178	ML-1	THCA
785	930083	RO82-W-1	THCA
786	930299	TT	THCA
787	1240223	TT2609-C02	THCA

Supplementary Table S3. List of 229 unique GDSC compounds used for training and validation of CDRscan

No	Name	Targets	Target pathway	PubCHEM ID	Sample size
**1**	681640	WEE1, CHEK1	Cell cycle	10384072	
**2**	(5Z)-7-Oxozeaenol	TAK1	Other, kinases	9863776	951
**3**	5-Fluorouracil	Antimetabolite (DNA & RNA)	Other	3385	971
**4**	A-443654	AKT1, AKT2, AKT3	PI3K/MTOR signaling	10172943	426
**5**	A-770041	LCK, FYN	Other, kinases	9549184	427
**6**	Afatinib	ERBB2, EGFR	EGFR signaling	10184653	952
**7**	AICA Ribonucleotide	AMPK agonist	Metabolism	65110	883
**8**	AKT inhibitor VIII	AKT1, AKT2, AKT3	PI3K/MTOR signaling	10196499	982
**9**	Alectinib	ALK	RTK signaling	49806720	977
**10**	Amuvatinib	KIT, PDGFRA, FLT3	Other, kinases	11282283	973
**11**	AR-42	HDAC1	Chromatin histone acetylation	6918848	968
**12**	AS601245	JNK1, JNK2, JNK2	JNK and p38 signaling	10109823	928
**13**	AS605240	PI3Kgamma	PI3K/MTOR signaling	5289247	978
**14**	AT-7519	CDK1, CDK2, CDK4, CDK6, CDK9	Cell cycle	11338033	979
**15**	Avagacestat	Amyloid beta20, Amyloid beta40	Other	46883536	980
**16**	Axitinib	PDGFR, KIT, VEGFR	RTK signaling	6450551	888
**17**	AZ628	BRAF	ERK MAPK signaling	11676786	429
**18**	AZD6482	PI3Kbeta	PI3K/MTOR signaling	44137675	941
**19**	AZD7762	CHEK1, CHEK2	Cell cycle	11152667	892
**20**	AZD8055	MTORC1, MTORC2	PI3K/MTOR signaling	25262965	880
**21**	BAY-61-3606	SYK	Other, kinases	10200390	928
**22**	Belinostat	HDAC1	Chromatin histone acetylation	6918638	951
**23**	Bexarotene	Retinioic X receptor (RXR) agonist	Other	82146	919
**24**	BI-2536	PLK1, PLK2, PLK3	Cell cycle	11364421	425
**25**	Bicalutamide	AR	Hormone-related	2375	930
**26**	BIX02189	MEK5, ERK5	ERK MAPK signaling	46931012	978
**27**	BMS-345541	IKK1, IKK2	Other, kinases	9813758	983
**28**	BMS-509744	ITK	Other	20635522	427
**29**	BMS-536924	IGF1R, IR	IGFR signaling	10390396	426

Continues on the next page

No	Name	Targets	Target pathway	PubCHEM ID	Sample size
**30**	BMS-754807	IGF1R, IR	IGFR signaling	24785538	927
**31**	Bortezomib	Proteasome	Protein stability and degradation	387447	427
**32**	Bosutinib	SRC, ABL, TEC	Other, kinases	5328940	894
**33**	Bryostatin 1	PKC	Other, kinases	5280757	926
**34**	BX796	TBK1, PDK1 (PDPK1), IKK, AURKB, AURKC	Other	10077147	888
**35**	BX-912	PDK1 (PDPK1)	PI3K/MTOR signaling	11754511	981
**36**	Cabozantinib	VEGFR, MET, RET, KIT, FLT1, FLT3, FLT4, TIE2,AXL	Other, kinases	25102847	978
**37**	Camptothecin	TOP1	DNA replication	104842	892
**38**	CAY10603	HDAC1, HDAC6	Chromatin histone acetylation	24951314	971
**39**	CCT007093	PPM1D	Other	2314623	962
**40**	CCT-018159	HSP90	Protein stability and degradation	5327091	939
**41**	Cetuximab	EGFR	EGFR signaling	85668777	903
**42**	CGP-082996	CDK4	Cell cycle	24825971	426
**43**	CGP-60474	CDK1,CDK2,CDK5,CDK7,CDK9, PKC	Cell cycle	644215	426
**44**	CHIR-99021	GSK3A, GSK3B	WNT signaling	9956119	950
**45**	CI-1040	MEK1, MEK2	ERK MAPK signaling	6918454	881
**46**	Cisplatin	DNA crosslinker	DNA replication	84691	893
**47**	CMK	RSK2	ERK MAPK signaling	16663089	426
**48**	CP466722	ATM	Genome integrity	44551660	983
**49**	CP724714	ERBB2	EGFR signaling	9874913	980
**50**	Crizotinib	MET, ALK, ROS1	RTK signaling	11626560	434
**51**	CUDC-101	HDAC1-10, EGFR, ERBB2	Other	24756910	960
**52**	CX-5461	RNA Polymerase 1	Other	25257557	974
**53**	Cyclopamine	SMO	Other	several	422
**54**	Cytarabine	Antimetabolite	DNA replication	6253	889
**55**	Dabrafenib	BRAF	ERK MAPK signaling	44462760	903
**56**	Dacinostat	HDAC1	Chromatin histone acetylation	6445533	932
**57**	Dactolisib	PI3K (Class 1), MTORC1, MTORC2	PI3K/MTOR signaling	11977753	881
**58**	Daporinad	NAMPT	Metabolism	6914657	935
**59**	Dasatinib	ABL, SRC, Ephrins, PDGFR, KIT	Other	3062316	423
**60**	DMOG	HIF-PH	Metabolism	560326	937

Continues on the next page

No	Name	Targets	Target pathway	PubCHEM ID	Sample size
**61**	Docetaxel	Microtubule stabiliser	Mitosis	148124	893
**62**	Doramapimod	p38, JNK2	JNK and p38 signaling	156422	877
**63**	Doxorubicin	Anthracycline	DNA replication	31703	931
**64**	EHT-1864	RAC1, RAC2, RAC3	Cytoskeleton	9938202	964
**65**	Elesclomol	HSP90	Protein stability and degradation	300471	892
**66**	Embelin	XIAP	Apoptosis regulation	3218	932
**67**	Entinostat	HDAC1, HDAC3	Chromatin histone acetylation	4261	428
**68**	Enzastaurin	PKCB	Other, kinases	176167	981
**69**	Epothilone B	Microtubule stabiliser	Mitosis	448013	931
**70**	Erlotinib	EGFR	EGFR signaling	176870	393
**71**	Etoposide	TOP2	DNA replication	36462	940
**72**	Fedratinib	JAK2	Other, kinases	16722836	979
**73**	FH535	PPARgamma, PPARdelta	WNT signaling	3463933	928
**74**	FMK	RSK	Other, kinases	none	839
**75**	FR-180204	ERK1, ERK2	ERK MAPK signaling	11493598	978
**76**	FTI-277	Farnesyl-transferase (FNTA)	Other	3005532	940
**77**	Gefitinib	EGFR	EGFR signaling	123631	889
**78**	Gemcitabine	Pyrimidine antimetabolite	DNA replication	60750	926
**79**	GNF-2	BCR-ABL	ABL signaling	5311510	424
**80**	GSK1070916	AURKA, AURKC	Mitosis	46885626	952
**81**	GSK1904529A	IGF1R, IR	IGFR signaling	25124816	931
**82**	GSK269962A	ROCK1, ROCK2	Cytoskeleton	16095342	966
**83**	GSK319347A	IKK	Other	11626927	429
**84**	GSK429286A	ROCK1, ROCK2	Cytoskeleton	11373846	983
**85**	GSK650394	SGK2, SGK3	Other	25022668	922
**86**	GSK690693	AKT1, AKT2, AKT3	PI3K/MTOR signaling	16725726	978
**87**	GW-2580	CSF1R	RTK signaling	11617559	980
**88**	GW441756	NTRK1	RTK signaling	9943465	890
**89**	GW843682X	PLK1	Cell cycle	9826308	427
**90**	HG-5-113-01	LOK, LTK, TRCB, ABL(T315I)	Other	none	519
**91**	HG-5-88-01	EGFR, ADCK4	Other, kinases	none	518
**92**	HG6-64-1	BRAF	ERK MAPK signaling	53302361	936

Continues on the next page

No	Name	Targets	Target pathway	PubCHEM ID	Sample size
**93**	I-BET-762	BRD2, BRD3, BRD4	Chromatin other	46943432	975
**94**	Idelalisib	PI3Kdelta	PI3K/MTOR signaling	11625818	982
**95**	Imatinib	ABL, KIT, PDGFR	RTK signaling	5291	436
**96**	IOX2	EGLN1	Other	54685215	967
**97**	IPA-3	PAK1	Cytoskeleton	521106	930
**98**	Ispinesib Mesylate	KSP	Mitosis	6450816	982
**99**	JNK Inhibitor VIII	JNK	JNK and p38 signaling	11624601	886
**100**	JNK-9L	JNK2, JNK3	JNK and p38 signaling	25222038	940
**101**	JQ1	BRD2, BRD3, BRD4, BRDT	Chromatin other	46907787	950
**102**	JW-7-24-1	LCK	Other, kinases	none	980
**103**	JW-7-52-1	MTOR	PI3K/MTOR signaling	49836027	411
**104**	KIN001-244	PDK1 (PDPK1)	Other, kinases	56965967	977
**105**	KIN001-260	IKKB	Other	10451420	977
**106**	KIN001-266	MAP3K8	Other, kinases	44143370	978
**107**	KIN001-270	CDK9	Cell cycle	66577006	978
**108**	KU-55933	ATM	Genome integrity	5278396	887
**109**	Lapatinib	ERBB2, EGFR	EGFR signaling	208908	422
**110**	Lenalidomide	CRBN	Protein stability and degradation	216326	893
**111**	Lestauritinib	FLT3, JAK2, NTRK1, NTRK2, NTRK3	Other, kinases	126565	892
**112**	LFM-A13	BTK	Other, kinases	54676905	930
**113**	Linifanib	VEGFR1, VEGFR2, VEGFR3, CSF1R, FLT3, KIT	RTK signaling	11485656	980
**114**	Linsitinib	IGF1R	IGFR signaling	11640390	927
**115**	Luminespib	HSP90	Protein stability and degradation	10096043	921
**116**	Masitinib	KIT, PDGFRA, PDGFRB	Other, kinases	10074640	979
**117**	Methotrexate	Antimetabolite	DNA replication	126941	892
**118**	MG-132	Proteasome, CAPN1	Protein stability and degradation	462382	427
**119**	Midostaurin	PKC, PPK, FLT1, c-FGR, others	Other	several	941
**120**	Mitomycin-C	DNA crosslinker	DNA replication	5746	932
**121**	MK-2206	AKT1, AKT2	PI3K/MTOR signaling	46930998	864
**122**	Motesanib	VEGFR, RET, KIT, PDGFR	RTK signaling	11667893	887

Continues on the next page

No	Name	Targets	Target pathway	PubCHEM ID	Sample size
**123**	MPS-1-IN-1	MPS1	Mitosis	25195352	976
**124**	Navitoclax	BCL2, BCL-XL, BCL-W	Apoptosis regulation	24978538	888
**125**	NG-25	TAK1, MAP4K2	Other, kinases	53340664	977
**126**	Nilotinib	ABL	ABL signaling	644241	843
**127**	NPK76-II-72-1	PLK3	Cell cycle	none	978
**128**	NSC-207895	MDM4	p53 pathway	42640	971
**129**	NSC-87877	SHP-1 (PTPN6), SHP-2 (PTPN11)	Other	5459322	936
**130**	NU7441	DNAPK	Genome integrity	11327430	885
**131**	Nutlin-3a (-)	MDM2	p53 pathway	11433190	888
**132**	NVP-BHG712	EPHB4	Other	16747388	978
**133**	NVP-TAE684	ALK	RTK signaling	16038120	434
**134**	Obatoclax Mesylate	BCL2, BCL-XL, BCL-W, MCL1	Apoptosis regulation	11404337	923
**135**	Olaparib	PARP1, PARP2	Genome integrity	23725625	940
**136**	Omipalisib	PI3K (class 1), MTORC1, MTORC2	PI3K/MTOR signaling	25167777	980
**137**	OSI-027	MTORC1, MTORC2	PI3K/MTOR signaling	44224160	974
**138**	OSI-930	KIT	RTK signaling	9868037	979
**139**	OSU-03012	PDK1 (PDPK1)	Other, kinases	10027278	930
**140**	PAC-1	Procaspase-3, Procaspase-7	Apoptosis regulation	6753378	919
**141**	Paclitaxel	Microtubule stabiliser	Mitosis	36314	427
**142**	Palbociclib	CDK4, CDK6	Cell cycle	5330286	864
**143**	Parthenolide	HDAC1	Chromatin histone acetylation	7251185	429
**144**	Pazopanib	CSF1R, KIT, PDGFRA, PDGFRB	RTK signaling	10113978	928
**145**	PD0325901	MEK1, MEK2	ERK MAPK signaling	9826528	880
**146**	PD173074	FGFR1, FGFR3	RTK signaling	1401	888
**147**	Pelitinib	EGFR	EGFR signaling	6445562	979
**148**	Pevonedistat	NAE	Other	16720766	725
**149**	PF-4708671	S6K1	PI3K/MTOR signaling	51371303	950
**150**	PF-562271	FAK, FAK2	Cytoskeleton	11713159	921
**151**	PFI-1	BRD4	Chromatin other	71271629	969
**152**	PHA-665752	MET	RTK signaling	10461815	435
**153**	PHA-793887	CDK2, CDK7, CDK5	Cell cycle	46191454	980

Continues on the next page

No	Name	Targets	Target pathway	PubCHEM ID	Sample size
**154**	Phenformin	Biguanide agent	Other	8249	969
**155**	PI-103	PI3Kalpha, DAPK3, CLK4, PIM3, HIPK2	PI3K/MTOR signaling	9884685	969
**156**	Pictilisib	PI3K (class 1)	PI3K/MTOR signaling	17755052	919
**157**	PIK-93	PI3Kgamma	PI3K/MTOR signaling	6852167	978
**158**	Piperlongumine	Induces reactive oxygen species	Other	637858	965
**159**	PLX-4720	BRAF	ERK MAPK signaling	24180719	955
**160**	Ponatinib	ABL, PDGFRA, VEGFR2, FGFR1, SRC, TIE2, FLT3	RTK signaling	24826799	940
**161**	Pyrimethamine	Dihydrofolate reductase (DHFR)	Other	4993	429
**162**	QS11	ARFGAP1	Other	4263900	931
**163**	Quizartinib	FLT3	RTK signaling	24889392	979
**164**	Rapamycin	MTORC1	PI3K/MTOR signaling	5384616	389
**165**	Refametinib	MEK1, MEK2	ERK MAPK signaling	44182295	910
**166**	RO-3306	CDK1	Cell cycle	44450571	888
**167**	Rucaparib	PARP1, PARP2	Genome integrity	9931953	964
**168**	Ruxolitinib	JAK1, JAK2	Other, kinases	25126798	982
**169**	Salubrinal	EIF2A	Other	5717801	424
**170**	Saracatinib	ABL, SRC	RTK signaling	10302451	436
**171**	SB216763	GSK3A, GSK3B	WNT signaling	176158	785
**172**	SB-505124	ALK4, ALK5	RTK signaling	9858940	966
**173**	SB52334	ALK5	RTK signaling	9967941	977
**174**	SB590885	BRAF	ERK MAPK signaling	11316960	868
**175**	Seliciclib	CDK2, CDK7, CDK9	Cell cycle	160355	422
**176**	Selisistat	SIRT1	Chromatin histone acetylation	5113032	974
**177**	Selumetinib	MEK1, MEK2	ERK MAPK signaling	10127622	956
**178**	Sepantronium bromide	BIRC5	Apoptosis regulation	11178236	945
**179**	Serdemetan	MDM2	p53 pathway	11609586	961
**180**	SGC0946	DOT1L	Chromatin histone methylation	56962337	944
**181**	Shikonin	not defined	Other	5208	937
**182**	SL0101	RSK, AURKB, PIM1, PIM3	Other	10459196	872

Continues on the next page

No	Name	Targets	Target pathway	PubCHEM ID	Sample size
**183**	SN-38	TOP1	DNA replication	104842	968
**184**	SNX-2112	HSP90	Protein stability and degradation	24772860	970
**185**	Sorafenib	PDGFR, KIT, VEGFR, RAF	RTK signaling	216239	431
**186**	STF-62247	Autophagy inducer	Other	704473	975
**187**	S-Trityl-L-cysteine	KIF11	Mitosis	76044	425
**188**	Sunitinib	PDGFR, KIT, VEGFR, FLT3, RET, CSF1R	RTK signaling	5329102	426
**189**	T0901317	LXR, FXR	Other	447912	972
**190**	TAK-715	p38alpha, p38beta	JNK and p38 signaling	9952773	983
**191**	Talazoparib	PARP1, PARP2	Genome integrity	44819241	952
**192**	Tamoxifen	ESR1	Hormone-related	2733526	966
**193**	Tanespimycin	HSP90	Protein stability and degradation	6505803	889
**194**	Temozolomide	DNA alkylating agent	DNA replication	5394	944
**195**	TGX221	PI3Kbeta	PI3K/MTOR signaling	9907093	427
**196**	Thapsigargin	SERCA	Other	446378	914
**197**	Tipifarnib	Farnesyl-transferase (FNTA)	Other	159324	928
**198**	Tivozanib	VEGFR1, VEGFR2, VEGFR3	RTK signaling	9911830	978
**199**	Tozasertib	AURKA, AURKB, AURKC, others	Mitosis	5494449	421
**200**	TPCA-1	IKK2	Other, kinases	9903786	978
**201**	Trametinib	MEK1, MEK2	ERK MAPK signaling	11707110	930
**202**	Tretinoin	Retinoic acid	Other	444795	883
**203**	Tubastatin A	HDAC1, HDAC6, HDAC8	Chromatin histone acetylation	53394750	974
**204**	TW 37	BCL2, BCL-XL, MCL1	Apoptosis regulation	11455910	963
**205**	UNC0638	G9a and GLP methyltransferases	Chromatin histone methylation	46224516	972
**206**	UNC1215	L3MBTL3	Chromatin other	57339144	945
**207**	Veliparib	PARP1, PARP2	Genome integrity	11960529	893
**208**	Vinblastine	Microtubule destabiliser	Mitosis	6710780	893
**209**	Vinorelbine	Microtubule destabiliser	Mitosis	5311497	941
**210**	Vismodegib	SMO	Other	24776445	893
**211**	VNLG/124	HDAC,RAR	Chromatin histone acetylation	24894414	974

Continues on the next page

No	Name	Targets	Target pathway	PubCHEM ID	Sample size
**212**	Vorinostat	HDAC inhibitor Class I, IIa, IIb, IV	Chromatin histone acetylation	5311	894
**213**	VX-11e	ERK2	ERK MAPK signaling	11634725	977
**214**	VX-702	p38	JNK and p38 signaling	10341154	890
**215**	WH-4-023	SRC, LCK	Other, kinases	11844351	424
**216**	WHI-P97	JAK3	Other, kinases	3796	978
**217**	WIKI4	TNKS1, TNKS2	WNT signaling	2984337	975
**218**	WZ-1-84	BMX	Other, kinases	49821040	426
**219**	WZ3105	SRC, ROCK2, NTRK2, FLT3, IRAK1, others	Other	none	979
**220**	XAV939	TNKS1, TNKS2	WNT signaling	2726824	956
**221**	XMD8-85	ERK5, BET	Other	46844147	423
**222**	XMD8-92	MAPK7	Other, kinases	46843772	520
**223**	Y-39983	ROCK	Cytoskeleton	9810884	978
**224**	YK-4-279	RNA helicase A	Other	44632017	821
**225**	YM201636	PYKFYVE	Other	9956222	978
**226**	Zibotentan	Endothelin-1 receptor (EDNRA)	Other	9910224	977
**227**	Z-LLNle-CHO	gamma-secretase	Other	16760646	426
**228**	ZM447439	AURKA, AURKB	Mitosis	9914412	836
**229**	ZSTK474	PI3K (class 1)	PI3K/MTOR signaling	11647372	982

Supplementary Table S4. Five-fold cross validation of CDRscan.

**Model**	R^2					
	**Fold 0**	**Fold 1**	**Fold 2**	**Fold 3**	**Fold 4**	**Mean**
**CDRscan**						
master	0.844	0.848	0.846	0.851	0.845	0.847
fully connected	0.843	0.850	0.846	0.846	0.850	0.847
shallow	0.846	0.849	0.847	0.847	0.850	0.848
tanh	0.846	0.852	0.846	0.847	0.849	0.848
unified	0.848	0.850	0.843	0.846	0.850	0.847
